# Phenolic signals for prehaustorium formation in *Striga hermonthica*

**DOI:** 10.3389/fpls.2022.1077996

**Published:** 2022-12-06

**Authors:** Natsumi Aoki, Songkui Cui, Chiharu Ito, Kie Kumaishi, Shungo Kobori, Yasunori Ichihashi, Satoko Yoshida

**Affiliations:** ^1^ Division of Biological Science, Graduate School of Science and Technology, Nara Institute of Science and Technology, Ikoma, Japan; ^2^ Department of Economic Plants and Biotechnology, Yunnan Key Laboratory for Wild Plant Resources, Kunming Institute of Botany, Chinese Academy of Sciences, Kunming, China; ^3^ RIKEN BioResource Research Center, Tsukuba, Japan

**Keywords:** striga, prehaustorium, phenolics, quinone, parasitic plants

## Abstract

*Striga hermonthica* is a root parasitic plant that causes considerable crop yield losses. To parasitize host plants, parasitic plants develop a specialized organ called the haustorium that functions in host invasion and nutrient absorption. The initiation of a prehaustorium, the primitive haustorium structure before host invasion, requires the perception of host-derived compounds, collectively called haustorium-inducing factors (HIFs). HIFs comprise quinones, phenolics, flavonoids and cytokinins for *S. hermonthica*; however, the signaling pathways from various HIFs leading to prehaustorium formation remain largely uncharacterized. It has been proposed that quinones serve as direct signaling molecules for prehaustorium induction and phenolic compounds originating from the host cell wall are the oxidative precursors, but the overlap and distinction of their downstream signaling remain unknown. Here we show that quinone and phenolic-triggered prehaustorium induction in *S. hermonthica* occurs through partially divergent signaling pathways. We found that ASBr, an inhibitor of acetosyringone in virulence gene induction in the soil bacterium *Agrobacterium*, compromised prehaustorium formation in *S. hermonthica*. In addition, LGR-991, a competitive inhibitor of cytokinin receptors, inhibited phenolic-triggered but not quinone-triggered prehaustorium formation, demonstrating divergent signaling pathways of phenolics and quinones for prehaustorium formation. Comparisons of genome-wide transcriptional activation in response to either phenolic or quinone-type HIFs revealed markedly distinct gene expression patterns specifically at the early initiation stage. While quinone DMBQ triggered rapid and massive transcriptional changes in genes at early stages, only limited numbers of genes were induced by phenolic syringic acid. The number of genes that are commonly upregulated by DMBQ and syringic acid is gradually increased, and many genes involved in oxidoreduction and cell wall modification are upregulated at the later stages by both HIFs. Our results show kinetic and signaling differences in quinone and phenolic HIFs, providing useful insights for understanding how parasitic plants interpret different host signals for successful parasitism.

## Introduction

The Orobanchaceae family contains root parasitic plants with a spectrum of parasitic lifestyles: facultative parasites, obligate hemiparasites, and obligate holoparasites (Westwood et al., 2010). Of the family members, the obligate hemiparasite *Striga* spp. And holoparasites *Phelipanche* spp. And *Orobanche* spp. Infect various staple crops, such as rice, sorghum, carrot and sunflower, leading to huge economic losses *via* yield reduction (Rodenburg et al., 2016; Nosratti et al., 2020). Parasitic plants infect hosts by a multicellular organ, the haustorium, which specializes in host attachment, invasion, interspecific vascular connection and nutrient deprivation (Cui et al., 2016; Yoshida et al., 2016; Cui et al., 2020; Wakatake et al., 2020). Haustorium development involves multiple steps including prehaustorium formation, tissue penetration and vascular connection with the host, and requires intense signaling interactions between the host and parasitic plants in each of these stages (Clarke et al., 2019; Furuta et al., 2021; Mutuku et al., 2021).

Orobanchaceae parasitic plants generally require host-derived compounds, called haustorium-inducing factors (HIFs) to initiate prehaustorium formation (Goyet et al., 2019). In obligate parasites, the radical tip undergoes rapid deformation toward a terminal prehaustorium upon exposure to HIFs by terminating root meristematic activity and redirecting to cell expansion (Keyes et al., 2000; Xiao et al., 2022). HIF signals are thus perceived and transduced into cell reprogramming resulting in morphological changes in the root tip toward the host-parasitizing organ. On the other hand, facultative parasites do not terminate root growth, instead; they form lateral haustoria above the root meristematic zone leading to the formation of multiple haustoria on the lateral side of a root (Tomilov et al., 2005; Cui et al., 2016). Despite differences in the haustorium initiation sites and their morphology, both facultative and obligate parasites can respond to the same HIFs, *i.e.*, facultative parasite *Phtheirospermum japonicum* and the obligate parasite *S. hermonthica* initiate prehaustorium formation by a similar set of monolignols and quinones that bear certain structural properties (Cui et al., 2018). Diverged HIF recognition also exists among three parasitic forms: *S. hermonthica* but not *P. japonicum* reacts to cytokinins (CKs) as HIFs (Aoki et al., 2022), whereas the holoparasite *Phelipanche ramosa* responds primarily to CKs (Goyet et al., 2017), and forms prehaustoria against only high concentration of quinones at millimolar range (Fernandez-Aparicio et al., 2021), reflecting reduced ability of holoparasites in sensing phenolics and quinones.

*Striga* spp. perceive various types of HIFs, including phenolics, quinones, flavonoids and CKs, which are secondary metabolites or phytohormones (Keyes et al., 2000; Cui et al., 2018; Aoki et al., 2022). A quinone molecule, DMBQ (2,6-dimethyl-1,4-benzoquinone) was the first HIF identified in sorghum tissue, a natural host of *S. hermonthica* (Chang and Lynn, 1986), and was later found to be potent against many hemiparasites, including the model parasitic plants *P. japonicum* and *Triphysaria* spp. Thus, DMBQ has been widely used for *in vitro* prehaustorium induction assays (Matvienko et al., 2001a and 2001b Ishida et al., 2016). In Arabidopsis, DMBQ is sensed *via* the plasma membrane-localized LRR receptor kinase CANNOT RESPOND TO DMBQ 1 (CARD1, also known as HPCA1), leading to a Ca^2+^ increase in the cytosol (Laohavisit et al., 2020). DMBQ also induces rapid activation of mitogen-activated protein kinases (MAPK) and the expression of genes related to stress and defense responses in Arabidopsis, conferring higher resistance to pathogens (Laohavisit et al., 2020). Transcriptomic analyses in the roots of *P. japonicum* and *T. versicolor* after DMBQ treatments revealed similar gene expression profiles as those in Arabidopsis, in which genes related to stress responses and oxidoreduction-related genes were strongly upregulated (Matvienko et al., 2001a; Cohen et al., 2004; Ishida et al., 2016). Although direct binding of quinones to the CARD1 receptor has not been clarified, given that CARD1 homologs CADL1/2/3 in either *Striga asiatica* or *P. japonicum* all rescued Ca^2+^-increase deficiency in Arabidopsis *card1*, it is currently assumed that quinone triggers prehaustorium formation *via* CARD1 homologs (Laohavisit et al., 2020).

Cell wall-related phenolics, including syringic acid (SyA), acetosyringone (AS) and vanillic acid, were reported to induce prehaustorium in *S. hermonthica* (Lynn and Chang, 1990; Cui et al., 2018). These phenolic-type HIFs commonly have a hydroxyl group at position 4 of a benzene ring and methoxy groups at positions 3 and/or 5. It appears that the number of methoxy groups affects prehaustorium induction activity in *S. hermonthica* and *P. japonicum* (Cui et al., 2018). The hosts’ lignin biosynthesis pathway partially but not exclusively contributes to HIF production, indicating the existence of multiple routes for the origin of HIFs, in line with the ability of sensing diverse HIFs in *Striga*. It has been proposed that phenolics act as the precursors of quinones that trigger prehaustorium formation (Keyes et al., 2001), as SyA and sinapic acid are oxidatively converted to DMBQ *in vitro* by peroxidases presumably produced by parasitic plants (Kim et al., 1998). Although plants produce various phenolic compounds as metabolites or as rhizosphere secreted compounds, in general, phenolic compounds have not been considered signaling molecules for plants, except for a few examples, such as salicylic acid. In addition, plant-derived phenolic compounds are known as signaling molecules for microbes. In soil-borne bacteria *Rhizobium radiobacter* (synom. *Agrobacterium tumefaciens*), phenolic compounds, including AS from plants, trigger the expression of *Virulence* (*Vir*) genes to facilitate DNA transfer into plant genomes (Lee et al., 1995). In this case, transmembrane histidine kinase VirA is known as the phenolic sensor because mutation or swapping of the *VirA* gene altered phenolic sensing in *Agrobacterium*, yet its direct binding to phenolics was not observed (Lee et al., 1995; Fang et al., 2015). Interestingly, the structural specificity of phenolic compounds that trigger vir gene expression in Agrobacterium is, to some extent, similar to HIFs for parasitic plants (Lynn and Chang, 1990). It is a tempting hypothesis that, similar to bacteria, plants can directly perceive phenolic compounds, but no evidence has been provided thus far.

CKs, the essential phytohormones governing multiple processes of growth and development, induce prehaustoria in *S. asiatica* and *S. hermonthica* at the nanomolar scale. CKs exhibit higher potency than other HIFs by approximately more than two magnitudes (Keyes et al., 2000; Aoki et al., 2022). CKs are perceived by ARABIDOPSIS HISTIDINE KINASES (AHK2, 3, 4) involved in two-component regulatory systems, and the signals are transduced by downstream response regulators. Our recent study demonstrated that LGR-991, a CK receptor antagonist that directly binds to AHKs inhibited prehaustorium formation by CKs, indicating the importance of direct CK perception by AHKs for prehaustorium induction (Aoki et al., 2022). Because LGR-991 did not inhibit DMBQ-induced prehaustorium formation, CK perception is not necessary for DMBQ signaling. In addition, CKs and DMBQ induced distinct marker genes during prehaustorium formation (Aoki et al., 2022). Furthermore, TFBQ, an inhibitor of prehaustorium induction, inhibited both CK- and DMBQ- induced prehaustorium formation, suggesting that CKs are perceived independently from quinone while their signaling pathways converge downstream for prehaustorium induction.

Formation of prehaustorium is the first transition step from autotrophic to heterotrophic lifestyles for parasitic plants and thus represents a key step to control parasitic weeds. The complexity of HIFs reflects the capacity to parasitize a wider spectrum of hosts, particularly certain obligate parasitic plants in need of immediate parasitization after germination. To better understand the molecular pathways underlying prehaustorium induction, we investigated the interaction of phenolic signaling with quinone signaling by performing inhibitor assays and transcriptome analyses in *S. hermonthica*. Our results reveal distinct responses to phenolics and quinones during prehaustorium formation, suggesting the possibility of the presence of the phenolic signaling pathway in plants.

## Materials and methods

### Plant materials

The plant materials and growth conditions used were previously reported in Aoki et al. (2022). Briefly, *S. hermonthica* seeds of approximately 50-100 mg were sterilized with 20% commercial bleach solution (Kao Ltd., Tokyo, Japan; final concentration of sodium hypochlorite was approximately 0.6%) for 5 min, washed at least 5 times with sterilized water, poured into petri dishes with moist glass fiber filter paper (GE Healthcare UK Ltd., Little Chalfont, UK), and preconditioned for 1 week at 25°C in the dark. Ten nanomolar (+)-strigol (Hirayama and Mori, 1999) was directly added to *Striga* seeds in a petri dish and incubated for 24 h at 25°C in the dark to induce germination. Germinated seedlings were subjected to the inhibitor assay described below. The rice cultivar *Oryza sativa* cv. Koshihikari and *A. thaliana* ecotype Columbia (Col-0) were used for the exudate experiment. Root exudates of rice and Arabidopsis were collected as previously reported (Aoki et al., 2022)

### Inhibitor assay for prehaustorium induction

Germinated *S. hermonthica* seedlings were transferred to 96-well plates (Iwaki, Shizuoka, Japan) with each well containing HIFs with or without chemical inhibitors in a total volume of 100 µl. Chemical stocks of HIFs and inhibitors were initially prepared using DMSO as the solvent and added to each well according to the final working concentrations. Approximately 20~40 seedlings were placed in each well. The plates were kept at 25°C for 24 h in the dark followed by quantification of prehaustorium formation by observation under a stereomicroscope (Zeiss, Oberkochen, Germany, Stemi-2000). Prehaustorium induction rates were measured by the ratio of the number of *S. hermonthica* with prehaustorium versus the total number of seedlings in each well. For each treatment, three wells were measured as three replicates.

### Chemicals

The chemicals used in this study were obtained from the following providers: DMBQ (Sigma−Aldrich, St Louis, MO, USA), acetosyringone (Tokyo Chemical Industry Co., Tokyo, Japan), syringic acid (Sigma−Aldrich), kinetin (Sigma−Aldrich), 2-isopentenyladenine (2iP), tetrafluoro-1,4-benzoquinone (TFBQ, Sigma−Aldrich), alpha-bromoacetosyringone (ASBr, Toronto Research Chemicals, North York, Canada), 6-benzylaminopurine (BA, Wako Pure Chemical Co., Osaka, Japan), *trans*-zeatin (Wako Pure Chemical Co.) and thidiazuron (Wako Pure Chemical Co.). LGR-991 (Nisler et al., 2010) was a kind gift from Dr. Lukáš Spíchal at the Czech Advanced Technology and Research Institute.

### qRT−PCR

After treatment with water or syringic acid for 1, 6 or 24 h, *S. hermonthica* seedlings were collected for RNA extraction and a subsequent qRT−PCR assay was performed using a previously described method and primers (Aoki et al., 2022).

### Transcriptome analysis of *S. hermonthica*

One-day-old *S. hermonthica* seedlings were treated with or without 10 µM DMBQ or SyA and harvested at 1, 3, 6, 12, 18 and 24 hr after treatments (**Supplemental Table 1**). As biological replicates, at least three different sample sets were prepared at each time point. RNA sequence libraries were constructed with the breath adapter directional sequencing (BrAD)-seq method according to a previously published protocol (Townsley et al., 2015). Sequence reads were obtained by an Illumina 2500 sequencer for 151 cycles and paired-ends. After trimming adaptor sequences and low quality nucleotides using Trimmomatic (ver0.39) (Bolger et al., 2014) and fastqc (https://www.bioinformatics.babraham.ac.uk/projects/fastqc/), the sequence reads were mapped to the *S. hermonthica* genome assembly (Qiu et al., 2022) using HiSAT2 (Kim et al., 2019). The mapped reads were counted with the featurecounts function in the R subread library (https://sourceforge.net/projects/subread/files/subread-1.5.2/), and the differentially expressed genes were detected by edgeR (Robinson et al., 2009). The genes with low expression levels (total cpm per sample less than 4.5) were removed from the analysis. The expression values are shown in transcripts per million (tpm). The fold change in the expression value was calculated by dividing the DMBQ- or SyA- treated sample tpm by the mock treatment expression value. To avoid division by zero, 10^-5^ was added to the expression values of each sample before calculation. Self-organized mapping (SOM) clustering was performed for the fold change values using Kohonen (Wehrens and Buydens, 2007). GO enrichment analysis was conducted by AgriGO (Tian et al., 2017) using the homologous (blast top hit) gene from *S. asiatica* proteins (Yoshida et al., 2019).

## Results

### Prehaustorium induction in *S. hermonthica* by phenolic compounds

**Figure 1:**
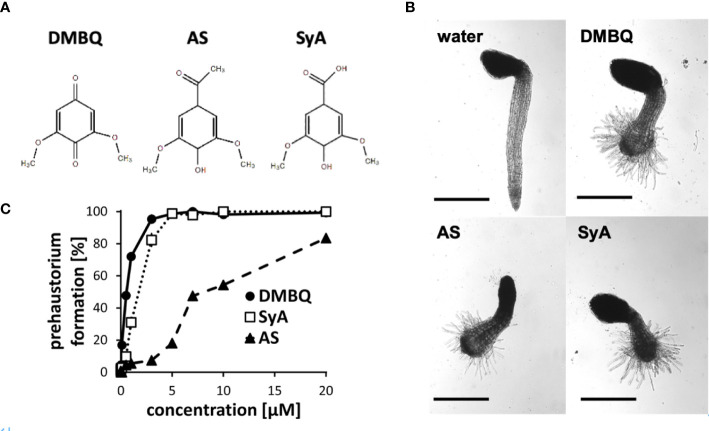
Prehaustorium induction by phenolic compounds acetosyringone and syringic acids. **(A)** Chemical structures of HIFs. DMBQ: a quinone. Acetosyringone (AS) and syringic acid (SyA): the phenolics. **(B)** Representative images of *S. hermonthica* seedings treated without (water) or with HIFs for 24 h upon germination. **(C)** Prehaustorium inducing activity of DMBQ (10 µM), AS (50 µM) and SyA (20 µM) after 24 h of treatment. Data represents mean ± SE with three replicates. Scale bars = 500 µm.

The phenolic HIFs syringic acid (SyA) and acetosyringone (AS) (**Figure 1A**) were previously reported to induce prehaustoria at 1~10 µM in *P. japonicum* and *S. hermonthica* (Cui et al., 2018; Wada et al., 2019). To compare their prehaustorium-inducing activities with quinone DMBQ, we applied SyA and AS to *S. hermonthica* seedlings. One day after treatments, *S. hermonthica* seedlings formed prehaustoria with a swollen root tip covered with haustorial hairs similar to DMBQ-induced prehaustorium (**Figure 1B**). To further investigate the prehaustorium-inducing activity of phenolic compounds, we conducted a kinetic experiment using various concentrations of the compounds. The kinetics showed a concentration-dependent linear increase in the prehaustorium induction rate by all compounds (**Figure 1C**). Whereas SyA induced prehaustorium in 100% of *S. hermonthica* seedlings at 5 µM, AS exhibited approximately 80% at even 20 µM, illustrating the higher potency of SyA compared to AS. These results indicate that the prehaustorium-inducing activity of phenolic compounds can be vary depending on the chemical properties of the groups at the para position of the phenol ring. Compared to these phenolic compounds, DMBQ displayed higher activity, consistent with a previous study (Wada et al., 2019).

### Effects of an acetosyringone inhibitor on prehaustorium induction

**Figure 2:**
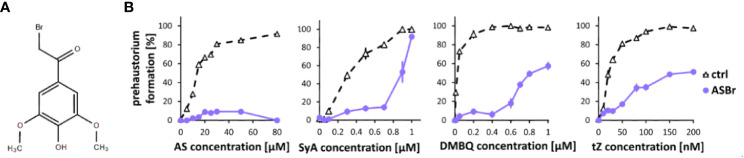
Effects of ASBr on prehaustorium induction triggered by syringic acid, quinone or cytokinin. **(A)** Chemical structure of α-bromoacetosyringone (ASBr), an inhibitor of AS in inducing virA-mediated vir genes in Agrobacterium for tumor induction. **(B)** S. hermonthica seedlings were treated by AS, SyA, DMBQ and a cytokinin *trans*-zeatin (tZ) at the concentrated indicated on the horizontal axis in the absence (ctrl) or presence of 10 µM ASBr for 24 h. Data represents mean ± SE with three replicates.

To investigate phenolic signaling in plants, we tested an inhibitor that is known to inhibit phenolic signaling in *Agrobacterium*. The gram-negative bacterium *A. tumefaciens* recognizes AS produced from wounded plant cell walls *via* VirA, a membrane-localized histidine kinase involved in the two-component regulatory system, to induce downstream *Vir* gene expression (Gelvin, 2000). While the direct binding of AS and VirA has not yet been confirmed, alpha-bromoacetosyringone (ASBr) (**Figure 2A**), a structural analog of AS (**Figure 2A**), can suppress AS-mediated *Vir* gene expression at a range of 1 to 10 µM (Hess et al., 1991; Lee et al., 1992). We found that application of 10 µM ASBr strongly inhibited prehaustorium formation by AS and to a lesser extent by SyA (**Figure 2B**), suggesting the presence of similar sensing mechanisms of AS in *S. hermonthica* and *Agrobacterium*. The solvent DMSO only was tested as control and no inhibitory effects was detected at the highest concentration (**Supplemental Figure 1**). To further explore the effects of ASBr on prehaustorium formation, we applied ASBr to DMBQ and *trans*-zeatin (tZ)-induced prehaustorium assays. The addition of 10 µM ASBr reduced prehaustorium formation by either DMBQ or cytokinins (CKs) in a concentration-dependent manner (**Figure 2B**; **Supplemental Figure 2**), suggesting that ASBr acts as a general prehaustorium inhibitor in *S. hermonthica*. Of note, however, the effect of ASBr appears to be strongest against AS among all HIFs tested, as shown by the lack of an increasing trend with increasing concentrations of AS. This may indicate that in addition to competitive inhibition of AS sensors, ASBr has general inhibitory properties for prehaustorium formation induced by various types of HIFs.

Because ASBr can suppress prehaustorium formation induced by various HIFs, this compound can be a potential candidate for a *Striga* control reagent. Thus, we tested whether ASBr suppresses prehaustorium formation induced by host root exudates derived from rice or Arabidopsis (**Supplemental Figure 3**). Application of ASBr suppressed prehaustorium induction by both rice and Arabidopsis root exudates. While ASBr almost completely suppressed rice exudate-induced prehaustorium formation, the effects on Arabidopsis exudate were moderate. These may reflect different composition of HIFs in root exudates from various species.

### Effects of quinone and cytokinin inhibitors on phenolic-induced prehaustorium formation

**Figure 3:**
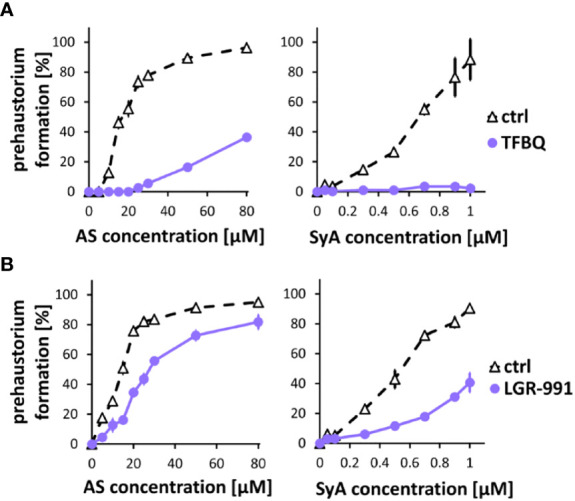
TFBQ and LGR-991 inhibit prehaustorium formation induced by phenolic compounds. S. hermonthica seedlings were treated with AS or SyA with indicated concentrations in the absence (ctrl) or presence of a quinone inhibitor TFBQ **(A)** and cytokinin receptor inhibitor LGR-991 **(B)**. 20 µM of TFBQ and 10 µM LGR-991 were used. Data represents mean ± SE with three replicates.

Following ASBr, which showed inhibitory effects on prehaustoria induced by various types of HIFs, we sought to explore inhibitors that could differentiate quinone- and phenolic-mediated signals. For this purpose, we chose two prehaustorium formation inhibitors, tetrafluoro-1,4-benzoquinone (TFBQ) and LGR-991. TFBQ is a known DMBQ inhibitor that can inhibit DMBQ-triggered prehaustorium formation in *S. asiatica*, *T. versicolor* and *P. japonicum* (Smith et al., 1996; Wang et al., 2019; Laohavisit et al., 2020) at a range of 0.1 to 100 µM. Our recent analysis, however, revealed that TFBQ at 20 µM can also inhibit CK-induced prehaustorium formation in *S. hermonthica* (Aoki et al., 2022), suggesting that TFBQ may be a general inhibitor of prehaustorium formation. LGR-991 is a CK analog and inhibits CK signaling by competitively binding to CK receptors (Nisler et al., 2010). In Arabidopsis study, 10 µM LGR-991 was shown to greatly inhibit activation of CK receptors *in vitro* and CK receptors mediated expression of downstream reporter *ARR5*. In *S. hermonthica*, 10 µM LGR-991 was previously shown to suppress CK-induced but not DMBQ-induced prehaustorium formation (Aoki et al., 2022). Similar to ASBr, we found that 20 µM TFBQ inhibited prehaustorium formation treated with either AS or SyA (**Figure 3A**). The inhibitory effect of TFBQ was the most profound against SyA, as determined by the absence of recovery in prehaustorium formation even at higher concentrations of SyA. On the other hand, increasing the concentration of AS partially recovered the induction in the presence of TFBQ. The inhibitory effect of TFBQ is thus stronger against SyA than AS, which is opposite to the effect of ASBr (**Figure 2B**). Such differences may reflect structural similarity to TFBQ of SyA compared to that of AS. To our surprise, treatment of 10 µM CK inhibitor LGR-991, which shows no effects on DMBQ-induced prehaustorium induction at the same concentration (Aoki et al., 2022), inhibited AS and SyA-induced prehaustorium formation (**Figure 3B**). The inhibition of prehaustorium formation was gradually recovered by increasing concentrations of either SyA or AS in the presence of LGR-991. Together, our data showed that ASBr and TFBQ compromise prehaustorium induction by all types of HIFs, while LGR-991 exerts an inhibitory effect exclusively on CKs and phenolics but not DMBQ. The effects of LGR-991 thus highlighted the difference in the quinone-induced and phenolic-induced signaling pathways for prehaustorium formation.

### Comparison of downstream gene regulation by syringic acid and DMBQ in *S. hermonthica* by transcriptome analysis

**Figure 4:**
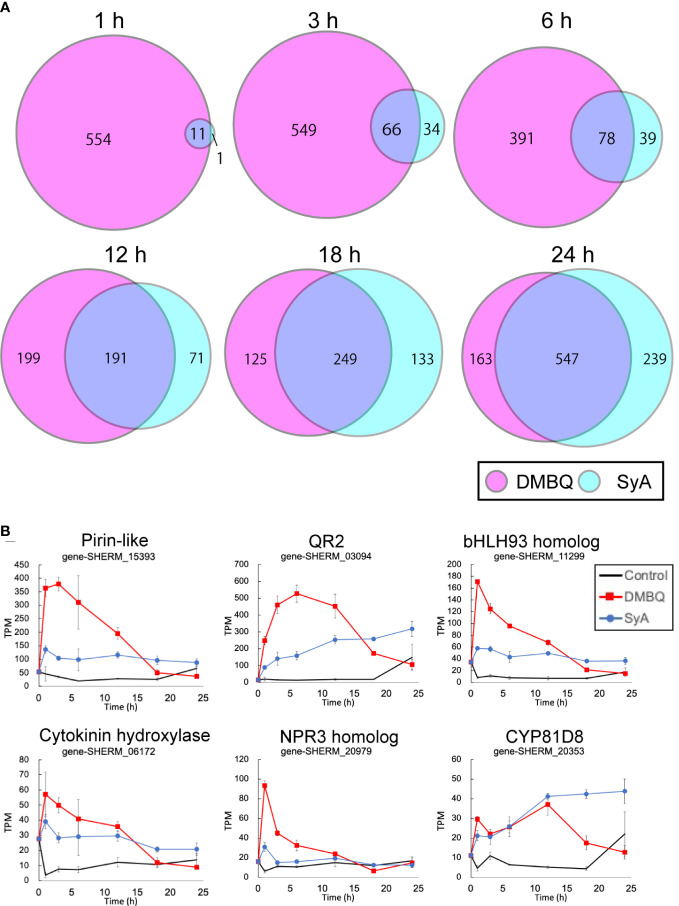
Number of transcripts upregulated at each time point after DMBQ or SyA treatments. **(A)** Venn diagrams show the number of transcripts (contigs) upregulated by DMBQ or SyA treatment at each time point. Differentially expressed genes were selected by edgeR based on the comparison of HIF treatment with water control. **(B)** Expression patterns of early core signaling genes at 1, 3, 6, 12, 18 and 24 h after treatment.

To understand genome-wide transcriptional regulation by phenolics and quinones, we surveyed transcriptional changes during prehaustorium formation in *S. hermonthica*. *S. hermonthica* seedlings treated with SyA or DMBQ for 0, 1, 3, 6, 12, 18 and 24 h were collected and subjected to RNA-seq analysis (**Supplemental Table 1**). Water-treated samples were collected at each time point as control. Differentially expressed genes (DEGs) were selected from each time point compared to water control samples (adjusted p value<0.05 by the Benjamini−Hochberg method, **Supplemental Tables 2, 3**). Interestingly, a large number of genes were upregulated by DMBQ, while only a small number of genes were upregulated by SyA (DMBQ vs. SyA:565 vs. 12) at 1 hour post induction (hpi) (**Figure 4A**). Eleven of 12 genes upregulated by SyA were also induced by DMBQ, representing commonly induced genes at the early time point. At 3 hpi, the number of upregulated DEGs by SyA treatment increased to 100 genes, while only a slight increase in the number of DEGs was observed in DMBQ (615). The DEG numbers continuously increased upon longer incubation with SyA to 24 hpi, when prehaustorium morphology is generally established. Compared to this gradually rising pattern, DMBQ-induced DEGs exhibited a transient decrease between 6 and 18 hpi. Overall, we found a continuous increase in a fraction of genes commonly regulated by DMBQ and SyA during prehaustorium formation (**Figure 4A**). A similar tendency was also observed for the downregulated DEGs by DMBQ and SyA; only one common commonly downregulated DEG was detected at 1 hpi and increased to 328 at 24 hpi (**Supplemental Figure 4**). These results suggest that only a set of several core genes is required for the onset of prehaustorium initiation, and along with morphological changes such as root tip swelling and haustorium hair proliferation, many genes are involved at the later stages.

The common genes induced at 1 hpi are possible early core genes for prehaustorium initiation (**Figure 4B**; **Supplemental Figure 5**). These genes include *QR2* and *Pirin*, which were previously reported to be involved in early prehaustorium formation stages in Orobanchaceae parasitic plants, indicating that our transcriptome analysis properly represents early molecular responses to HIF treatments. The early core gene set includes cytokinin hydroxylase (CYP735A), CYP81D8, defense-related NPR3 homolog, bHLH-type transcription factor, and an ABC transporter. Most of these genes presented higher levels of induction by DMBQ treatment than SyA treatment within 6 h and, conversely, decreased to lower levels than SyA treatment at 24 hpi.

### Temporal differences in DMBQ and SyA transcriptional responses

**Figure 5:**
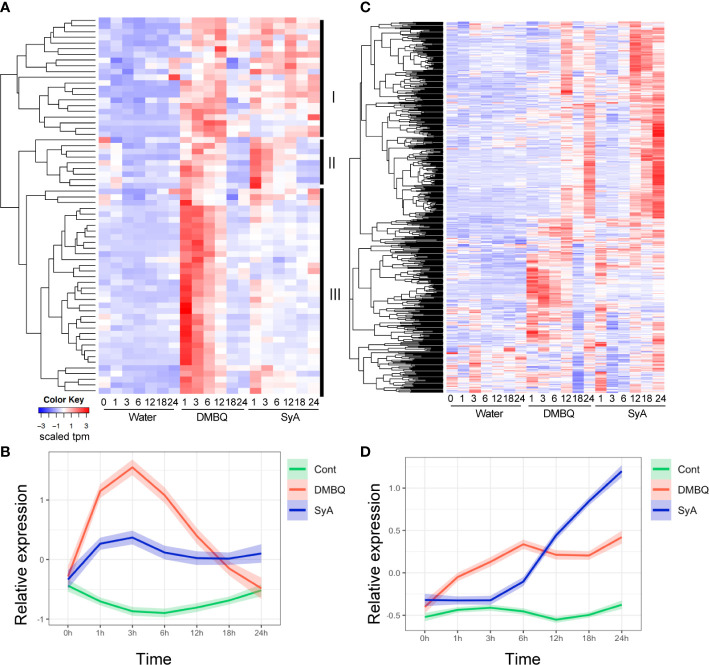
Expression patterns for 3-h and 24-h commonly upregulated contigs after DMBQ or SyA treatments. **(A)** Heatmap of relative expression values for 66 genes commonly upregulated by DMBQ or SyA at 3 h after treatment. Hierarchical clustering divide these transcripts into three large clusters I, II and III as labeled at the right. **(B)** The scaled expression values (tpm) of the 3-h commonly upregulated genes are shown by LOESS regression. **(C)** Heatmap of relative expression values for 534 genes commonly upregulated by DMBQ or SyA at 24 h after treatment. **(D)** The scaled expression values (tpm) of the 24-h commonly upregulated genes are shown by LOESS regression.

To further examine the temporal differences in gene induction by each HIF, we analyzed 66 genes commonly upregulated by DMBQ and SyA at 3 hpi (**Supplemental Table 4**). Hierarchical clustering of expression patterns indicates that these genes can be categorized into three clusters (**Figure 5A**). Cluster III contains genes that respond strongly to DMBQ at 1-3 hpi and decreased to the basal level at 18-24 hpi. On the other hand, these were induced moderately at 1-3 hpi by SyA treatment, and the expression levels were decreased to 24 hpi but maintained slightly higher levels compared to DMBQ at 24 hpi. Cluster II includes genes whose expression was induced 1-3 hpi by both DMBQ and SyA. The genes included in Cluster I showed induction at 3-6 hpi in both DMBQ and SyA, and their expression levels were decreased at 18-24 hpi in DMBQ samples but retained in SyA samples at these late time points (**Figure 5A**). Overall kinetic differences in gene expression by DMBQ and SyA were confirmed by LOESS regression analysis (**Figure 5B**). While DMBQ treatment induced rapid and higher transcript accumulation and peaked out before 24 h, SyA treatment showed relatively lower levels of gene induction at early stages that were maintained until the late stage of prehaustorium formation (**Figure 5B**). Similarly, 547 genes commonly upregulated at 24 hpi showed an early peak pattern by DMBQ, while slower and gradual increases eventually reached higher expression levels at 24 hpi by SyA (**Figures 5C, D**; **Supplemental Table 5**). These analyses indicate the distinct gene activation properties of these HIFs quick and strong gene induction by DMBQ, and slower but continuous gene induction by SyA.

To validate the occurrence of delayed gene expression by SyA, we analyzed the expression of *QR2*, *PIRIN*, *YUCCA3* and *EXPB1*, which were previously reported to be induced at the early time point upon DMBQ treatment in *S. hermonthica* (Aoki et al., 2022), after SyA treatment for 1, 6 and 24 h using qRT−PCR. Although substantial upregulation occurred at 1 and 6 h after SyA treatments compared to the water control, significant induction was observed at 24 h for all these genes (**Supplemental Figure 6**), further confirming the RNA-seq results. We also tested the expression of these genes by AS, another phenolic HIF. Although induction of these genes tends to be observed in AS treatment at a similar time point as SyA, significant upregulation of these genes was not confirmed (**Supplemental Figure 6**). This may reflect the lower prehaustorium induction activity of AS. Taken together, our results show that whereas DMBQ triggered marked transcriptional changes at the initial stage, SyA resulted in progressive expressional increases along prehaustorium formation.

### Global time course gene regulation during prehaustorium formation

To understand time-course transcriptional changes during phenolic- or quinone-induced prehaustorium formation, we performed clustering analysis of 2684 DEGs in SyA or DMBQ compared to the water control (**Supplemental Table 6**). Principal component analysis (PCA) showed that PC1 contributed 29.7%, PC2 contributed 21.0%, PC3 contributed 12.0%, PC4 contributed 7.7%, PC5 contributed 6.5%, and PC6 contributed 6.2%, resulting in a cumulative contribution rate of 83.2% through PC6 (**Supplemental Figure 7**). PC1 and PC4 represent changes in genes over time, while PC2, PC3 and PC5 reflect changes induced by DMBQ and SyA treatments. These results suggest that gene expression changes are associated primarily with the exposure time and with the type of HIFs. Using the self-organized mapping (SOM) clustering method, DEGs were classified into 150 groups based on their expression patterns (**Supplemental Figure 8A**) and further into 10 clusters by hierarchal clustering (**Supplemental Figures 8B, C**, **Supplemental Table 6**). PCA and color code mapping showed reasonable separation of each cluster, indicating successful clustering in our analysis (**Supplemental Figure 8D**).

**Figure 6:**
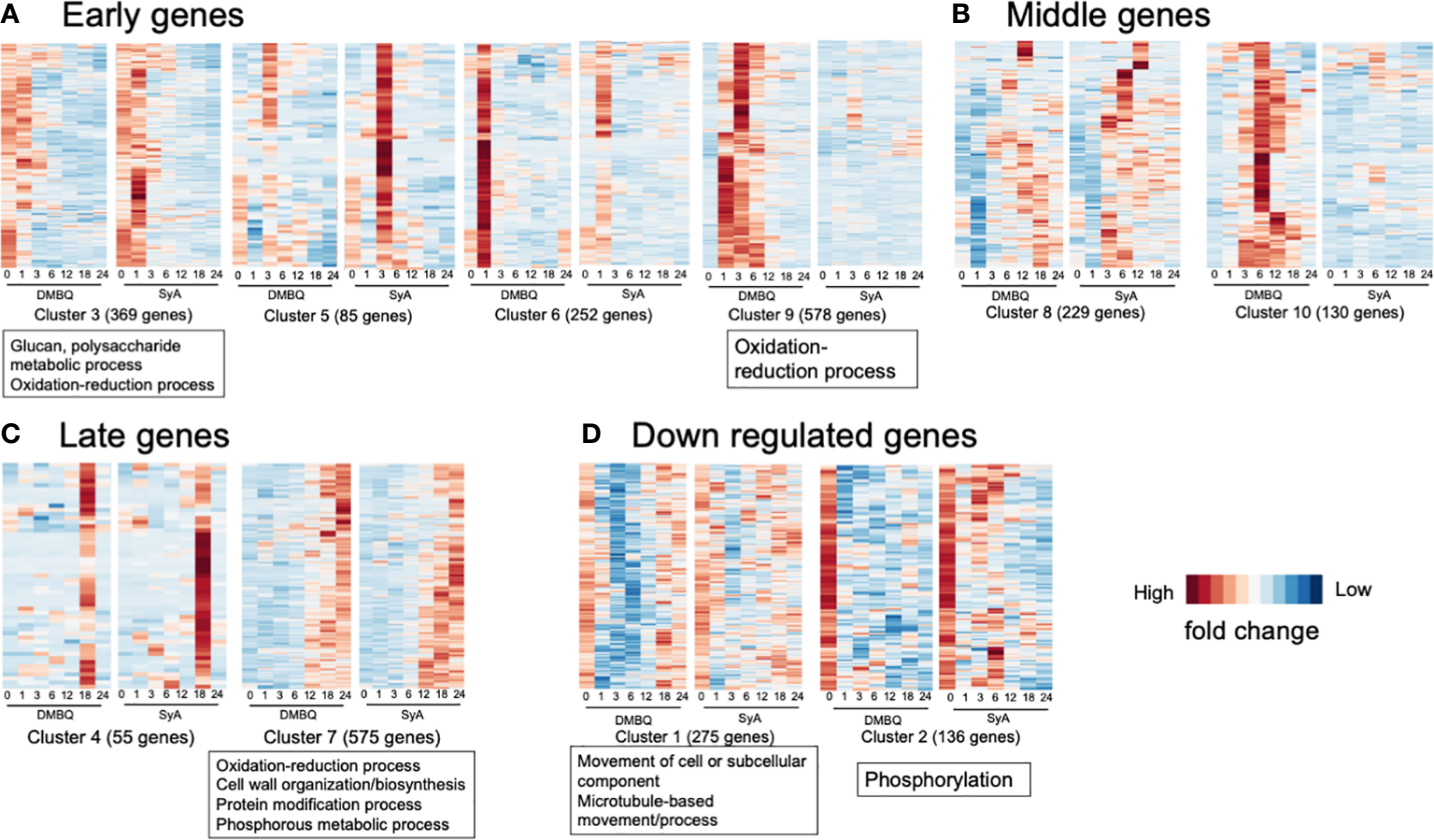
Clusters and GO terms for genes expressed at the early, middle and late stages of prehaustorium formation. **(A)** Clusters displaying genes differentially induced at 1 or 3 h after treatment of DMBQ or syringic acid. **(B)** Clusters displaying genes differentially induced at least at 6 or 12 h after treatment of DMBQ or syringic acid. **(C)** Clusters displaying genes differentially induced at least at 18 or 24 h after treatment of DMBQ or syringic acid. **(D)** Clusters displaying genes downregulated after treatment of DMBQ or syringic acid. Representative enriched GO terms for each cluster are shown in squares under heatmaps.

To classify the functions of DEGs along with different stages of prehaustorium formation, we dissected the prehaustorium formation process into early (1,3 hpi), middle (6, 12 hpi) and late (18, 24 hpi) stages. Early responsive DEGs fell in Clusters 3, 5, 6, and 9. Among these, Cluster 9 includes genes specifically responding to DMBQ while Clusters 3, 5 and 6 include genes responsive to both HIFs. Genes categorized to Cluster 3 were upregulated at 1 hpi and quickly downregulated after 3 hpi. Clusters 5 and 6 included genes strongly induced at 3 hpi and 1 hpi with stronger responses to SyA and DMBQ, respectively (**Figure 6A**). To analyze the functional classification of DEGs, Gene Ontology (GO) enrichment analysis was performed. GO analysis showed that DEGs in Cluster 9 were functionally enriched in the oxidation−reduction process, indicating dramatic changes in cellular redox status by DMBQ but not by SyA (**Figure 6A**; **Supplemental Table 7**). Cluster 3 showed GO enrichment for the terms glucan and polysaccharide metabolic process and oxidation-reduction process, representing early cell wall modification and redox regulation as early common responses to DMBQ and SyA (**Figure 6A**; **Supplemental Table 7**). At the middle stage, 299 DEGs were commonly upregulated by DMBQ and SyA treatments (Cluster 8), and 130 DEGs were specifically induced by DMBQ (Cluster 10), indicating that DMBQ-specific responses still occur at 6 hpi (**Figure 6B**; **Supplemental Table 7**). Despite the large number of genes classified into these clusters, no enriched GO terms were found in these clusters.

A total of 630 DEGs were classified as late-stage genes and showed induction by both HIFs. Cluster 7, containing a considerable number of DEGs (575) was functionally enriched in oxidation−reduction process, cell wall biosynthesis and organization, protein modification process, and phosphorous metabolic process, showing that these are linked with prehaustorium development (**Figure 6C**; **Supplemental Table 7**). These results indicate that both DMBQ and SyA trigger a large number of cell wall-related genes in common to establish the prehaustorium structure. Although most DEGs were upregulated, 411 DEGs were downregulated in the early stages (Clusters 1 and 2, **Figure 6D**). Both clusters showed sharp expression reduction by DMBQ at 1 hpi and more moderate downregulation by SyA (**Figure 6D**). Cluster 1 showed functional enrichment for the GO terms movement of cell or subcellular component, microtubule-based movement and process, and Cluster 2 showed enrichment in phosphorylation (**Figure 6D**; **Supplemental Table 7**). These results may reflect suppression of cell division at the root meristematic zone upon HIF treatment, resulting in reduced microtubule movement and phosphorylation of developmental signaling proteins.

### Expression of CK biosynthesis and signaling genes upon HIF treatments

**Figure 7:**
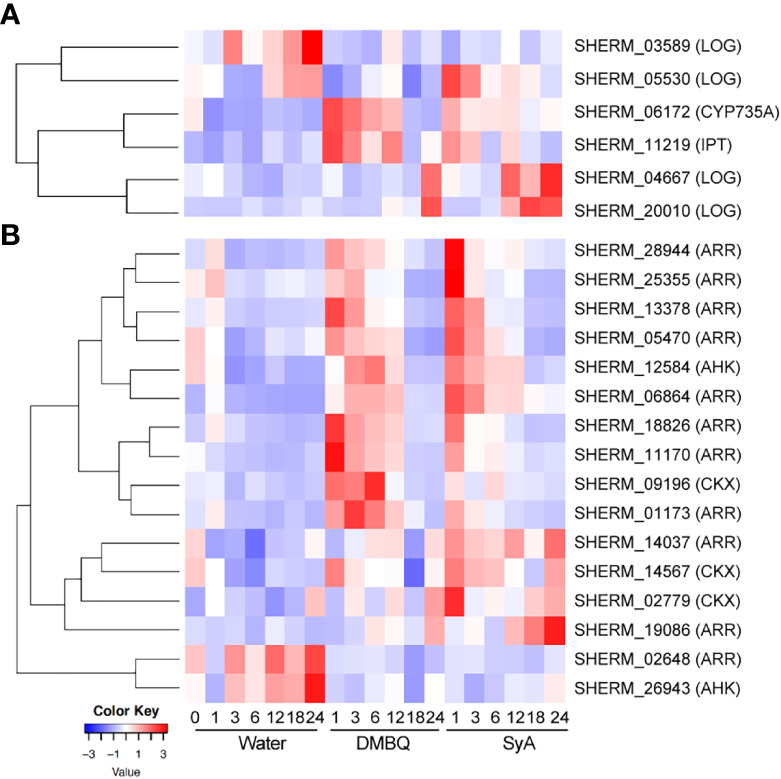
Expression of CK biosynthesis and signaling genes in response to DMBQ and SyA during prehaustorium formation. **(A)** The expression patterns of genes involved in CK biosynthesis (CYP735A, IPT, LOG) and **(B)** signaling and catabolism (AHK, ARR, CKX) were shown as heatmaps.

Since the CK inhibitor LGR-991 can suppress prehaustorium formation by phenolic compounds and early inducible genes include cytokinin dehydrogenase (CYP735A), which catalyzes hydroxylation of iP ribotides to tZ-type CK (Planas-Riverola et al., 2021), we hypothesized that CK biosynthesis may occur after phenolic HIF treatment. To test this possibility, CK biosynthesis and signaling genes were selected from the *S. hermonthica* genome annotation by reciprocal blast using Arabidopsis genes as queries (Qiu et al., 2022). As a biosynthesis gene, the *S. hermonthica* genome contains 1 CYP735A (cytokinin hydroxylase), 6 isopentenyltransferases (IPTs) and 10 LONELY GUY (LOGs) (**Supplemental Table 8**). Among them, 1 CYP735A, 1 IPT and 4 LOGs were detected as DEGs from the RNA-seq analysis of DMBQ- or SyA- treated *S. hermonthica* (**Supplemental Table 8**). An IPT gene (SHERM_11219) was upregulated together with CYP735A in DMBQ- and SyA- treated samples at early time points (**Figure 7A**; **Supplemental Figure 8**). This IPT gene is phylogenetically close to Arabidopsis IPT1, IPT4, IPT6 and IPT8, which were reported to be expressed in seeds (Nguyen et al., 2021). On the other hand, a LOG homolog (SHERM_05530) was induced at 1 hpi by SyA but not by DMBQ (**Figure 7A**). Other LOG genes also showed distinct expression patterns between DMBQ and SyA. SHERM-04667 and SHERM-20010, belonging to the Arabidopsis LOG8 and 9 clade, and LOG2 and 7 clade, respectively, increased their expression at 24 hpi in DMBQ and from 12-24 hpi in SyA (**Figure 7A**). Therefore, genes related to CK biosynthesis are induced to a greater extents by SyA than by DMBQ.

For CK signaling or catabolism genes, we searched for the genes encoding ShHK, ShRR and CKX, which are CK receptors, downstream response regulators, and CK hydrolases, respectively, in the *S. hermonthica* genome. Three ShHK, 5 ShHP, 21 ShRR and 14 CKX genes were found in the *S. hermonthica* genome, of which 2 ShHK, 11 ShRR and 3 CKX were detected as DEGs (**Supplemental Table 8**). One ShHK, 8 ShRR and one CKX gene were upregulated at early stages (1-6 hpi) by both DMBQ and SyA (**Figure 7B**; **Supplemental Figure 9**). ShRRs are generally classified into type-A and type-B, which function as negative and positive regulators in CK signaling, respectively (Kieber and Schaller, 2014). For the eight type-A ShRRs (SHERM_01173, 28944, 18826, 06864, 11170, 05470, 13378, and 25355) upregulated after treatment with both DMBQ and SyA, the upregulation by SyA tended to be stronger than that by DMBQ (**Figure 7B**; **Supplemental Figure 9**). Two type-B ShRRs (SHERM_14037 and 19086) and two CKXs (SHERM_14567 and 02779) were upregulated from early to late stages in SyA treatments, while the same genes were only slightly upregulated from 6 to 24 hpi in DMBQ (**Figure 7B**; **Supplemental Figure 9**). In addition, one ShRR (SHERM_02648) and one ShHK (SHERM_26943) were downregulated in the DMBQ and SyA treatments compared to the water control. These results suggest that DMBQ and SyA differentially regulate CK biosynthesis and signaling, which may link the different responses against CK perception inhibitors by DMBQ and SyA.

## Discussion

In the rhizosphere, phenolic compounds are often released by plant roots. Phenolic compounds are known to influence the soil-borne microbial community through their external antibacterial and antifungal activities or sometimes by attracting activity (Baetz and Martinoia, 2014). However, the influence of rhizosphere phenolic compounds on plants is not well understood. In Orobanchaceae parasitic plants, phenolics represent important host targeting signals for inducing prehaustoria. To date, the quinone HIF DMBQ has the main focus due to its high potency against various Orobanchaceae parasitic plants, and phenolics are considered precursors for quinone signals. Thus, limited information has been provided on how phenolics are perceived and induce prehaustorium. In this regard, we took a first step in characterizing phenolic signaling by inhibitor analysis and in terms of gene transcription in comparison to DMBQ signaling in *S. hermonthica*. In particular, it provides interesting insights into the unique transcriptional activation pattern between phenolics and quinones during prehaustorium formation. Moreover, we identified core early-signaling genes that might be minimally required for triggering prehaustorium induction and thus provide useful genetic information that could lead to a novel understanding of the signaling pathway and evolution of haustoria.

### Potential roles of ASBr in inhibiting prehaustorium formation in *Striga*

One of the significant findings in our study is the effect of ASBr, an inhibitor of VirA*-*mediated AS signal transduction in *Agrobacterium*, in inhibiting prehaustorium formation. Intriguingly, the suppressive effect was not only against AS and the structurally related phenolic SyA but also against quinone and CKs. The inhibitory effect of ASBr on all HIFs was unexpected but clearly indicates the converged signaling of phenolics, quinone and CKs during prehaustorium formation. The question is then what is the target of ASBr in *Striga*. In *Agrobacterium*, the cytoplasmic linker domain of VirA mediates phenol sensing (Turk et al., 1994; Fang et al., 2015; Swackhammer et al., 2022). ASBr is known as a specific and irreversible inhibitor of AS-induced *Vir* genes, acting in an AS competitive manner (Lee et al., 1992). However, the AS perception mechanisms of the VirA sensor are still under debate. Although structural modeling suggests the presence of a phenol binding site in the linker domain of VirA, direct binding of neither AS nor ASBr to VirA has been shown (Swackhammer et al., 2022). One possibility is that an adaptor protein that directly binds to AS mediates signal perception (Lee et al., 1992). Nevertheless, it was proposed that AS can directly or indirectly bind to a phenolic receptor, in which the carboxyl residue of phenolics activates the receptor by protonating the receptor surface that leads to conformational changes (Lee et al., 1992). In this model, replacement of a hydrogen with Br in the carboxyl residue of AS is assumed to interrupt the protonation. In parasitic plants, numerous lines of evidence suggest the importance of redox regulation involving ROS downstream of DMBQ perception; thus, the electron transfer system may play a crucial role in mediating prehaustorium induction (Matvienko et al., 2001a; Matvienko et al., 2001b; Bandaranayake et al., 2010; Ishida et al., 2016; Wada et al., 2019; Wang et al., 2019). Concomitantly, our transcriptomic data showed upregulation of genes bearing oxidoreduction activity by treatment with either syringic acid or DMBQ. Hence, we assume that ASBr may perturb the function of redox mediator(s)/receptors thus influencing the electron transfer system, which is a central hub of the prehaustorium induction system downstream of various HIFs. The suppressive effect of TFBQ, a DMBQ analog, on not only quinone but also phenolics and CKs (**Figure 3**) (Aoki et al., 2022) may also apply to this scenario, emphasizing converged signaling pathways at cellular oxidation and reduction processes involving redox regulation systems. Because ASBr effectively suppresses prehaustorium formation by rice root exudate, our findings present the potential utilization of ASBr as a *Striga* control reagent.

### Overlap and distinction of quinone and phenolic responses in *S. hermonthica*

Our transcriptome analysis revealed largely overlapping yet temporally distinct expression patterns between DMBQ and SyA responses. One of the significant differences was the immediate response upon exposure; compared to rapid and drastic transcriptional activation by DMBQ, only limited numbers of genes were induced by SyA at 1 hpi. The number of genes induced by SyA gradually increased and merged with DMBQ-responsive genes during prehaustorium development. Of note, despite differences in the expression patterns, more than half of the genes induced by SyA overlapped with the genes induced by DMBQ at each stage (**Figure 4**), indicating that transcriptional activation by SyA largely converges with DMBQ signaling alongside prehaustorium formation. Delayed induction of gene expression by SyA compared to DMBQ may indicate that syringic acid gradually undergoes conversion to DMBQ, as the previous model suggested (Keyes et al., 2001). It is still questionable, however, whether conversion could explain whole responses against SyA because SyA eventually induced higher responses than DMBQ, although the conversion rates were suggested to be 2-17% in *S. asiatica* seedlings or *in vitro* experiments (Kim et al., 1998). The gap can be filled if the assumption for the presence of a phenolic-specific pathway is taken into account. Indeed, the inhibitory effects of LGR-991 observed in SyA but not DMBQ (Aoki et al., 2022), as well as the distinct expression of CK-related genes, imply that phenolic compounds can drive signaling pathway distinct from those of quinones. Gradual conversion of SyA to DMBQ together with the contribution of presumed SyA-specific pathways may synergistically boost prehaustorial genes.

Another marked difference appears in the capacity of DMBQ, but not syringic acid, to induce a large number of genes within a short time frame (**Figure 4**). In Arabidopsis, DMBQ but not SyA was shown to induce a rapid increase in the level of cytosolic Ca^2+^ and activate the expression of genes related to defense and stress responses, conferring resistance toward pathogens in a CARD1-dependent manner (Laohavisit et al., 2020). Genes immediately induced by DMBQ in *S. hermonthica* are related to the oxidation−reduction process, indicating that the highly reactive nature of DMBQ may provoke oxidative stress responses unrelated to prehaustorium formation within this time frame.

To our surprise, SyA induced only 12 genes, 11 of which overlapped with DMBQ-induced genes at 1 h after treatment. Of these, *QR2* and *Pirin* were previously reported as early responsive genes to DMBQ in several parasitic Orobanchaceae (Bandaranayake et al., 2010; Bandaranayake et al., 2012; Ishida et al., 2017). The knockdown of *QR2* and *Pirin* leads to decreased prehaustorium formation in the facultative parasites *P. japonicum* and *T. versicolor*, respectively, suggesting the importance of these genes in prehaustorium formation (Bandaranayake et al., 2012; Ishida et al., 2017). The other early core genes include a homolog of transcription factor bHLH093, NPR3, and cytokinin hydroxylase. bHLH093 regulates nitrate transporters (NRTs) in response to light in *Arabidopsis* (Ruffel et al., 2021). Nitrogen is a primary target for parasitic plant nutrient acquisition from host plants and prehaustorium formation is suppressed by exogenous nitrogen (Kokla et al., 2022). Thus, prehaustorium formation processes may be adopted from nitrogen acquisition systems of autotrophic plants. NPR3 is known as a receptor component of the immune signal salicylic acid and negatively regulates immune responses (Ding et al., 2018). Induction of NPR3 homolog during prehaustorium formation may indicate another overlap between HIF response and immunity signals.

The late-stage genes commonly induced by DMBQ and SyA are enriched in GO terms related to cell wall organization and biosynthesis, suggesting that cell wall modification occurs during prehaustorium formation, presumably for rapid cell expansion (Yoshida et al., 2019). The large number of common genes in the late stage indicates that the cellular processes were similar between DMBQ and SyA responses at this stage, consistent with the morphological similarity of prehaustoria induced by different HIFs at 24 hpi.

### Interaction of phenolic signaling with cytokinin signaling

No detectable variation was found in the morphology of prehaustoria induced by phenolics, quinones or CKs, implying that their signaling must converge toward the same physiological output, at least in *S. hermonthica*. This was clearly supported by the inhibitory effect of ASBr and TFBQ on all of these HIFs (**Figure 3**) (Aoki et al., 2022). On the other hand, we found that LGR-991, a competitive inhibitor of CK that directly binds CK receptors, inhibited CKs, AS and SyA but not DMBQ (**Figure 3**) (Aoki et al., 2022). These results remarkably demonstrate the independence of quinone signaling transduction from CK perception and indicate that phenolic signaling is integrated into CK perception. CKs are perceived by several membrane-bound sensor histidine kinases (HKs) consisting of an extracellular CHASE domain, cytoplasmic HK domain and receiver domain, of which the CHASE domain biophysically binds CKs and LGR-911 (Nisler et al., 2010). The *S. asiatica* and *S. hermonthica* genomes contain 3 orthologs of Arabidopsis *AHKs* that bear those conserved domains (Yoshida et al., 2019). The effect of LGR-991 on both CKs and phenolics indicates that CK binding by CK receptors through the CHASE domain may be required for phenolic signal transduction. In this case, it would be plausible that phenolic acids induce CKs, thereby acting upstream of CKs for signal transduction. Coordinate induction of CK biosynthesis genes IPT, CYP735A1 and LOG may indicate activation of CK biosynthesis after SyA treatment. Interestingly, DMBQ induced that expression of IPT and CYP735A but not LOG at the early stage. Such a difference may cause different integration of CKs downstream of DMBQ and SyA, although how DMBQ and SyA differentially regulate CK biosynthesis genes remains unknown. The expression of CK signaling and catabolism genes also differed between DMBQ and SyA. The positive regulator of CK signaling B-type ShRR showed higher expression in SyA from the early to late time point, while it showed less induction in DMBQ. At early time points, A-type ShRRs, negative feedback regulators of CK signaling, are induced in both DMBQ and SyA with a tendency toward higher expression in SyA. Some of the CK catabolism genes CKXs also showed similar expression patterns. This may suggest that SyA induces temporal CK biosynthesis and response, but the signal is quickly downregulated by negative regulators. Further work is necessary to determine the crosstalk between phenolics and CKs through the measurement of CK levels in response to phenolics as well as quinone.

In conclusion, this study shows overlap and differences between quinone and phenolic signals for prehaustorium formation in *S. hermonthica*. The divergent signaling pathway may support the robustness of the initial transition step to become a parasite, which is crucial for the survival of obligate parasitic plants. Chemicals that attack the converged pathway from various HIFs, such as ASBr, could be useful components for future parasitic weed management.

## Data availability statement

The data presented in the study are deposited in the DDBJ repository, accession number DRA015133.

## Author contributions

NA, SC, and SY conceived this study. NA performed experiments, and CI and SY performed the bioinformatic analysis. KK, SK and YI constructed the RNA-seq libraries. SC and SY drafted the manuscript. All authors contributed to the article and approved the submitted version.

## References

[B1] Aoki N. Cui S. Yoshida S. (2022). Cytokinins induce prehaustoria coordinately with quinone signals in the parasitic plant *Striga hermonthica* . Plant Cell Physiol 63, 1446–1456. doi: 10.1093/pcp/pcac130 36112485

[B2] Baetz U. Martinoia E. (2014). Root exudates: the hidden part of plant defense. Trends Plant Sci. 19, 90–98. doi: 10.1016/j.tplants.2013.11.006 24332225

[B3] Bandaranayake P. C. G. Filappova T. Tomilov A. Tomilova N. B. Jamison-McClung D. Ngo Q.. (2010). A single-electron reducing quinone oxidoreductase is necessary to induce haustorium development in the root parasitic plant *Triphysaria* . Plant Cell 22, 1404–1419. doi: 10.1105/tpc.110.074831 20424175 PMC2879752

[B4] Bandaranayake P. C. G. Tomilov A. Tomilova N. B. Ngo Q. A. Wickett N. dePamphilis C. W.. (2012). The TvPirin gene is necessary for haustorium development in the parasitic plant *Triphysaria versicolor* . Plant Physiol. 158, 1046–1053. doi: 10.1104/pp.111.186858 22128136 PMC3271741

[B5] Bolger A. M. Lohse M. Usadel B. (2014). Trimmomatic: A flexible trimmer for illumina sequence data. Bioinformatics 30, 2114–2120. doi: 10.1093/bioinformatics/btu170 24695404 PMC4103590

[B6] Chang M. Lynn D. G. (1986). The haustorium and the chemistry of host recognition in parasitic angiosperms. J. Chem. Ecol. 12, 561–579. doi: 10.1007/BF01020572 24306796

[B7] Clarke C. R. Timko M. P. Yoder J. I. Axtell M. J. Westwood J. H. (2019). Molecular dialog between parasitic plants and their hosts. Annu. Rev. Phytopathol. 57, 279–299. doi: 10.1146/annurev-phyto-082718-100043 31226021

[B8] Cohen R. Suzuki M. R. Hammel K. E. (2004). Differential stress-induced regulation of two quinone reductases in the brown rot basidiomycete *Gloeophyllum trabeum* . Appl. Environ. Microbiol. 70, 324–331. doi: 10.1128/AEM.70.1.324-331.2004 14711659 PMC321286

[B9] Cui S. Kubota T. Nishiyama T. Ishida J. K. Shigenobu S. Shibata T. F.. (2020). Ethylene signaling mediates host invasion by parasitic plants. Sci. Adv. 6, eabc2385. doi: 10.1126/sciadv.abc2385 33115743 PMC7608805

[B10] Cui S. Wada S. Tobimatsu Y. Takeda Y. Saucet S. B. Takano T.. (2018). Host lignin composition affects haustorium induction in the parasitic plants *Phtheirospermum japonicum* and *Striga hermonthica* . New Phytol. 218, 710–723. doi: 10.1111/nph.15033 29498051

[B11] Cui S. Wakatake T. Hashimoto K. Saucet S. B. Toyooka K. Yoshida S.. (2016). Haustorial hairs are specialized root hairs that support parasitism in the facultative parasitic plant *Phtheirospermum japonicum* . Plant Physiol. 170, 1492–1503. doi: 10.1104/pp.15.01786 26712864 PMC4775136

[B12] Ding Y. Sun T. Ao K. Peng Y. Zhang Y. Li X.. (2018). Opposite roles of salicylic acid receptors NPR1 and article opposite roles of salicylic acid receptors NPR1 and NPR3 / NPR4 in transcriptional regulation of plant immunity. Cell 173, 1454–1467.e15. doi: 10.1016/j.cell.2018.03.044 29656896

[B13] Fang F. Lin Y. H. Pierce B. D. Lynn D. G. (2015). A *Rhizobium radiobacter* histidine kinase can employ both boolean and and or logic gates to initiate pathogenesis. ChemBioChem 16, 2183–2190. doi: 10.1002/cbic.201500334 26310519

[B14] Fernandez-Aparicio M. Masi M. Cimmino A. Evidente A. (2021). Effects of benzoquinones on radicles of *Orobanche* and *Phelipanche* species. Plants (Basel) 10, 746. doi: 10.3390/plants10040746 33920368 PMC8070214

[B15] Furuta K. M. Xiang L. Cui S. Yoshida S. (2021). Molecular dissection of haustorium development in Orobanchaceae parasitic plants. Plant Physiol. 186, 1424–1434. doi: 10.1093/plphys/kiab153 33783524 PMC8260117

[B16] Gelvin S. B. (2000). Agrobacterium and plant genes involved in T-DNA transfer and integration. Annu. Rev. Plant Physiol. Plant Mol. Biol. 51, 223–256. doi: 10.1146/annurev.arplant.51.1.223 15012192

[B17] Goyet V. Billard E. Pouvreau J. B. Lechat M. M. Pelletier S. Bahut M.. (2017). Haustorium initiation in the obligate parasitic plant *Phelipanche ramosa* involves a host-exudated cytokinin signal. J. Exp. Bot. 68, 5539–5552. doi: 10.1093/jxb/erx359 29069455 PMC5853424

[B18] Goyet V. Wada S. Cui S. Wakatake T. Shirasu K. Montiel G.. (2019). Haustorium inducing factors for parasitic Orobanchaceae. Front. Plant Sci. 10, 1056. doi: 10.3389/fpls.2019.01056 31555315 PMC6726735

[B19] Hess K. M. Dudley M. W. Lynn D. G. Joerger R. D. Binns A. N. (1991). Mechanism of phenolic activation of Agrobacterium virulence genes: development of a specific inhibitor of bacterial sensor/response systems. Proc. Natl. Acad. Sci. U.S.A. 88, 7854–7858. doi: 10.1073/pnas.88.17.7854 1909032 PMC52402

[B20] Hirayama K. Mori K. (1999). Plant bioregulators, 5 - synthesis of (+)-strigol and (+)-orobanchol, the germination stimulants, and their stereoisomers by employing lipase-catalyzed asymmetric acetylation as the key step. Eur. J. Org Chem. 1999, 2211–2217. doi: 10.1002/(SICI)1099-0690(199909)1999:9<2211::AID-EJOC2211>3.0.CO;2-O

[B21] Ishida J. K. Wakatake T. Yoshida S. Takebayashi Y. Kasahara H. Wafula E.. (2016). Local auxin biosynthesis mediated by a YUCCA flavin monooxygenase regulates haustorium development in the parasitic plant *Phtheirospermum japonicum* . Plant Cell 28, 1795–1814. doi: 10.1105/tpc.16.00310 27385817 PMC5006708

[B22] Ishida J. K. Yoshida S. Shirasu K. (2017). Quinone oxidoreductase 2 is involved in haustorium development of the parasitic plant *Phtheirospermum japonicum* . Plant Signal Behav. 12, e1319029. doi: 10.1080/15592324.2017.1319029 28498050 PMC5586360

[B23] Keyes W. J. O'Malley R. C. Kim D. Lynn D. G. (2000). Signaling organogenesis in parasitic angiosperms: Xenognosin generation, perception, and response. J. Plant Growth Regul. 19, 217–231. doi: 10.1007/s003440000024 11038229

[B24] Keyes W. J. Taylor J. V. Apkarian R. P. Lynn D. G. (2001). Dancing together. social controls in parasitic plant development. Plant Physiol. 127, 1508–1512. doi: 10.1104/pp.010753 11743095 PMC1540184

[B25] Kieber J. J. Schaller G. E. (2014). Cytokinins. Arabidopsis Book 12, e0168. doi: 10.1199/tab.0168 24465173 PMC3894907

[B26] Kim D. Kocz R. Boone L. Keyes W. J. Lynn D. G. (1998). On becoming a parasite: evaluating the role of wall oxidases in parasitic plant development. Chem. Biol. 5, 103–117. doi: 10.1016/S1074-5521(98)90144-2 9495831

[B27] Kim D. Paggi J. M. Park C. Bennett C. Salzberg S. L. (2019). Graph-based genome alignment and genotyping with HISAT2 and HISAT-genotype. Nat. Biotechnol. 37, 907–915. doi: 10.1038/s41587-019-0201-4 31375807 PMC7605509

[B28] Kokla A. Leso M. Zhang X. Simura J. Serivichyaswat P. T. Cui S. (2022). Nitrogen represses haustoria formation through abscisic acid in the parasitic plant *Phtheirospermum japonicum* . Nat. Commun. 13, 2976. doi: 10.1038/s41467-022-30550-x 35624089 PMC9142502

[B29] Laohavisit A. Wakatake T. Ishihama N. Mulvey H. Takizawa K. Suzuki T.. (2020). Quinone perception in plants *via* leucine-rich-repeat receptor-like kinases. Nature 587, 92–97. doi: 10.1038/s41586-020-2655-4 32879491

[B30] Lee K. Dudley M. W. Hess K. M. Lynn D. G. Joerger R. D. Binns A. N. (1992). Mechanism of activation of Agrobacterium virulence genes: identification of phenol-binding proteins. Proc. Natl. Acad. Sci. U.S.A. 89, 8666–8670. doi: 10.1073/pnas.89.18.8666 1528878 PMC49981

[B31] Lee Y. W. Jin S. Sim W. S. Nester E. W. (1995). Genetic evidence for direct sensing of phenolic compounds by the VirA protein of *Agrobacterium tumefaciens* . Proc. Natl. Acad. Sci. U.S.A. 92, 12245–12249. doi: 10.1073/pnas.92.26.12245 8618878 PMC40333

[B32] Lynn D. G. Chang M. (1990). Phenolic signals in cohabitation - implications for plant development. Annu. Rev. Plant Physiol. Plant Mol. Biol. 41, 497–526. doi: 10.1146/annurev.pp.41.060190.002433

[B33] Matvienko M. Torres M. J. Yoder J. I. (2001a). Transcriptional responses in the hemiparasitic plant *Triphysaria versicolor* to host plant signals. Plant Physiol. 127, 272–282. doi: 10.1104/pp.127.1.272 11553755 PMC117983

[B34] Matvienko M. Wojtowicz A. Wrobel R. Jamison D. Goldwasser Y. Yoder J. I. (2001b). Quinone oxidoreductase message levels are differentially regulated in parasitic and non-parasitic plants exposed to allelopathic quinones. Plant J. 25, 375–387. doi: 10.1046/j.1365-313x.2001.00971.x 11260494

[B35] Mutuku J. M. Cui S. Yoshida S. Shirasu K. (2021). Orobanchaceae parasite-host interactions. New Phytol. 230, 46–59. doi: 10.1111/nph.17083 33202061

[B36] Nguyen H. N. Lai N. Kisiala A. B. Emery R. J. N. (2021). Isopentenyltransferases as master regulators of crop performance: their function, manipulation, and genetic potential for stress adaptation and yield improvement. Plant Biotechnol. J. 19, 1297–1313. doi: 10.1111/pbi.13603 33934489 PMC8313133

[B37] Nisler J. Zatloukal M. Popa I. Doležal K. Strnad M. Spíchal L. (2010). Cytokinin receptor antagonists derived from 6-benzylaminopurine. Phytochemistry 71, 823–830. doi: 10.1016/j.phytochem.2010.01.018 20189204

[B38] Nosratti I. Sabeti P. Chaghamirzaee G. Heidari H. (2020). Weed problems, challenges, and opportunities in Iran. Crop Prot. 134, 104371. doi: 10.1016/j.cropro.2017.10.007

[B39] O'Malley R. C. Lynn D. G. (2000). Expansin message regulation in parasitic angiosperms: marking time in development. Plant Cell 12, 1455–1465. doi: 10.2307/3871142 10948262 PMC149115

[B40] Planas-Riverola A. Markaide E. Cano-Delgado A. I. (2021). New role for LRR-receptor kinase in sensing of reactive oxygen species. Trends Plant Sci. 26, 102–104. doi: 10.1016/j.tplants.2020.11.011 33309457

[B41] Qiu S. Bradley J. M. Zhang P. Chaudhuri R. Blaxter M. Butlin R. K.. (2022). Genome-enabled discovery of candidate virulence loci in *Striga hermonthica*, a devastating parasite of African cereal crops. New Phytol 239, 622–638. doi: 10.1111/nph.18305 PMC9795911 35699626

[B42] Robinson M. D. McCarthy D. J. Smyth G. K. (2009). edgeR: A bioconductor package for differential expression analysis of digital gene expression data. Bioinformatics 26, 139–140. doi: 10.1093/bioinformatics/btp616 19910308 PMC2796818

[B43] Rodenburg J. Demont M. Zwart S. J. Bastiaans L. (2016). Parasitic weed incidence and related economic losses in rice in Africa. Agric. Ecosyst. Environ. 235, 306–317. doi: 10.1016/j.agee.2016.10.020

[B44] Ruffel S. Chaput V. Przybyla-Toscano J. Fayos I. Ibarra C. Moyano T.. (2021). Genome-wide analysis in response to nitrogen and carbon identifies regulators for root AtNRT2 transporters. Plant Physiol. 186, 696–714. doi: 10.1093/plphys/kiab047 33582801 PMC8154064

[B45] Smith C. E. Ruttledge T. Zeng Z. O'Malley R. C. Lynn D. G. (1996). A mechanism for inducing plant development: the genesis of a specific inhibitor. Proc. Natl. Acad. Sci. U.S.A. 93, 6986–6991. doi: 10.1073/pnas.93.14.6986 11607691 PMC38921

[B46] Swackhammer A. Provencher E. A. P. Donkor A. K. Garofalo J. Dowling S. Garchitorena K.. (2022). Mechanistic analysis of the VirA sensor kinase in *Agrobacterium tumefaciens* using structural models. Front. Microbiol. 13. doi: 10.3389/fmicb.2022.898785 PMC9149312 35651496

[B47] Tian T. Liu Y. Yan H. You Q. Yi X. Du Z.. (2017). AgriGO v2.0: A GO analysis toolkit for the agricultural community 2017 update. Nucleic Acids Res. . 45, W122–W129. doi: 10.1093/nar/gkx382 28472432 PMC5793732

[B48] Tomilov A. A. Tomilova N. B. Abdallah I. Yoder J. I. (2005). Localized hormone fluxes and early haustorium development in the hemiparasitic plant *Triphysaria versicolor* . Plant Physiol. 138, 1469–1480. doi: 10.1104/pp.104.057836 15965023 PMC1176418

[B49] Townsley B. T. Covington M. F. Ichihashi Y. Zumstein K. Sinha N. R. (2015). BrAD-seq: Breath Adapter Directional sequencing: a streamlined, ultra-simple and fast library preparation protocol for strand specific mRNA library construction. Front. Plant Sci. 6, 1–11. doi: 10.3389/fpls.2015.00366 26052336 PMC4441129

[B50] Turk S. C. van Lange R. P. Regensburg-Tuink T. J. Hooykaas P. J. (1994). Localization of the VirA domain involved in acetosyringone-mediated Vir gene induction in *Agrobacterium tumefaciens* . Plant Mol. Biol. 25, 899–907. doi: 10.1007/BF00028884 8075405

[B51] Wada S. Cui S. Yoshida S. (2019). Reactive oxygen species (ROS) generation is indispensable for haustorium formation of the root parasitic plant *Striga hermonthica* . Front. Plant Sci. 10, 328. doi: 10.3389/fpls.2019.00328 30967886 PMC6438919

[B52] Wakatake T. Ogawa S. Yoshida S. Shirasu K. (2020). An auxin transport network underlies xylem bridge formation between the hemi-parasitic plant *Phtheirospermum japonicum* and host Arabidopsis. Development 147, dev187781. doi: 10.1242/dev.187781 32586973

[B53] Wang Y. Steele D. Murdock M. Lai S. Yoder J. (2019). Small-molecule screens reveal novel haustorium inhibitors in the root parasitic plant *Triphysaria versicolor* . Phytopathology 109, 1878–1887. doi: 10.1094/PHYTO-04-19-0115-R 31241407

[B54] Wehrens R. Buydens L. M. C. (2007). Self-and super-organizing maps in r: the kohonen package. J. Stat. Software 21, 19. doi: 10.18637/jss.v021.i05

[B55] Westwood J. H. Yoder J. I. Timko M. P. dePamphilis C. W. (2010). The evolution of parasitism in plants. Trends Plant Sci. 15, 227–235. doi: 10.1016/j.tplants.2010.01.004 20153240

[B56] Xiao T. T. Kirschner G. K. Kountche B. A. Jamil M. Savina M. Lube V.. (2022). A PLETHORA/PIN-FORMED/auxin network mediates prehaustorium formation in the parasitic plant *Striga hermonthica* . Plant Physiol. 189, 2281–2297. doi: 10.1093/plphys/kiac215 35543497 PMC9342978

[B57] Yoshida S. Cui S. Ichihashi Y. Shirasu K. (2016). The haustorium, a specialized invasive organ in parasitic plants. Annu. Rev. Plant Biol. 67, 643–667. doi: 10.1146/annurev-arplant-043015-111702 27128469

[B58] Yoshida S. Kim S. Wafula E. K. Tanskanen J. Kim Y. M. Honaas L.. (2019). Genome sequence of *Striga asiatica* provides insight into the evolution of plant parasitism. Curr. Biol. 29, 3041–3052.e4. doi: 10.1016/j.cub.2019.07.086 31522940

