# Supplementary material for: Phenolic signals for prehaustorium formation in Striga hermonthica
Source: Front Plant Sci. 2022 Dec 6;13:1077996. doi: 10.3389/fpls.2022.1077996 (PMC9767415; doi:10.3389/fpls.2022.1077996)
Supplement: Supplementary file 1 [file DataSheet_1.pdf]

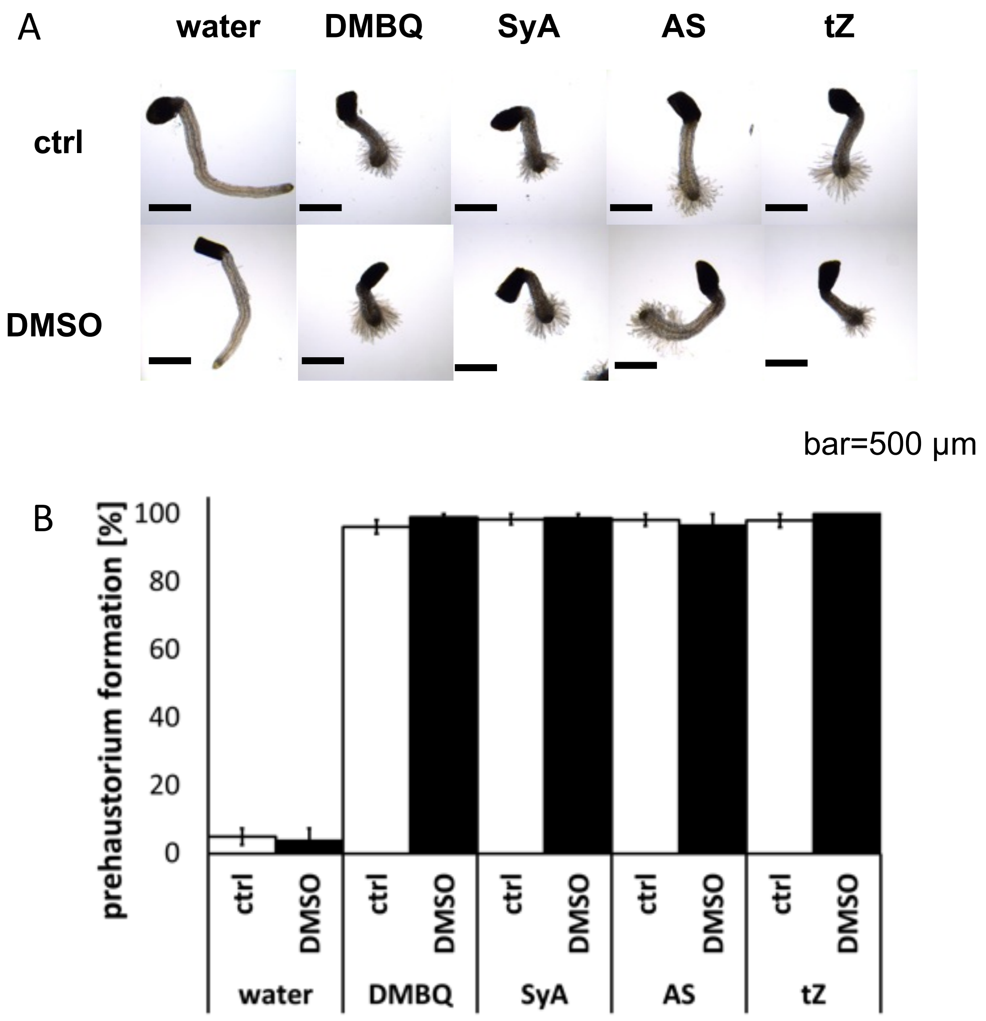

Supplemental Fig. 1. Effects of the solvent DMSO on prehaustorium formation in *S. hermonthica*. Prehaustorium formation was quantified after *S. hermonthica* seedlings were treated with various HIFs with or without 3.3% DMSO to confirm no inhibitory effects of DMSO. Photos of prehaustoria after each treatment are shown (A), and prehaustorium formation percentage (B) was calculated. Data represents mean±SE with three replicates.

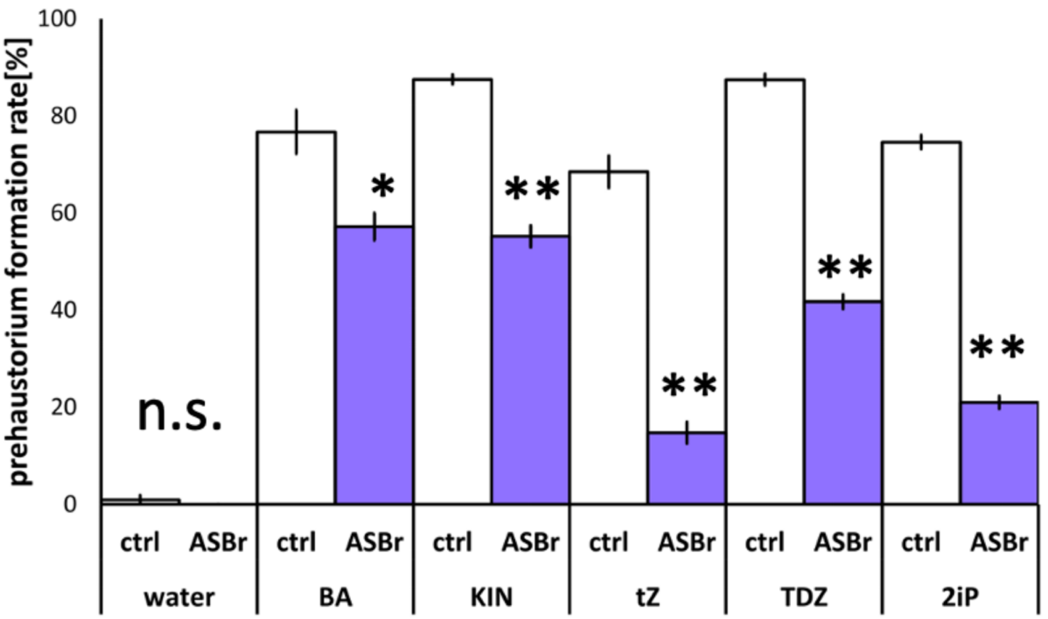

Supplemental Fig. 2. Effects of ASBr on prehaustorium formation treated with various cytokinins in *S. hermonthica*. Prehaustorium formation was quantified after *S. hermonthica* seedlings were treated with various cytokinins or water as a control in the absence (ctrl) or presence of 10 μM ASBr. 300 μM 6-benzylaminopurine（BA), 500 nM kinetin (KIN), 25 nM *trans*-zeatin (tZ), 100 nM N6-isopentenyladenine (2iP), and 25 nM of a synthetic derivative thidiazuron (TDZ) were used. Data represents mean±SE with three replicates. Asterisks indicate significant differences from the control (*t*-test, **: $p<0.01$, *: $0.01<p<0.05$).

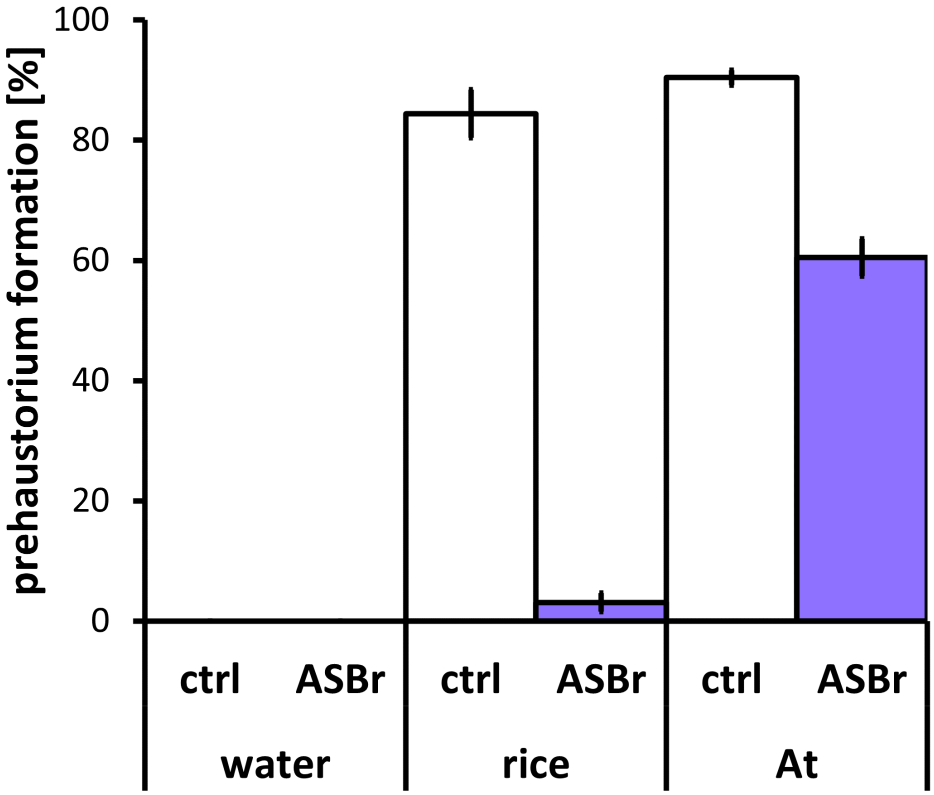

Supplemental Fig. 3. The effects of ASBr on the exudates of rice or Arabidopsis in inducing *S. hermonthica* prehaustoria. Prehaustorium formation was quantified after S. hermothica seedlings were treated with the exudates of rice or Arabidopsis (At) in the absence (ctrl) or presence of 10 μM ASBr. Data represents mean±SE with three replicates. Asterisks indicate significant differences from the control (*t*-test, **: $p<0.01$, *: $0.01<p<0.05$).

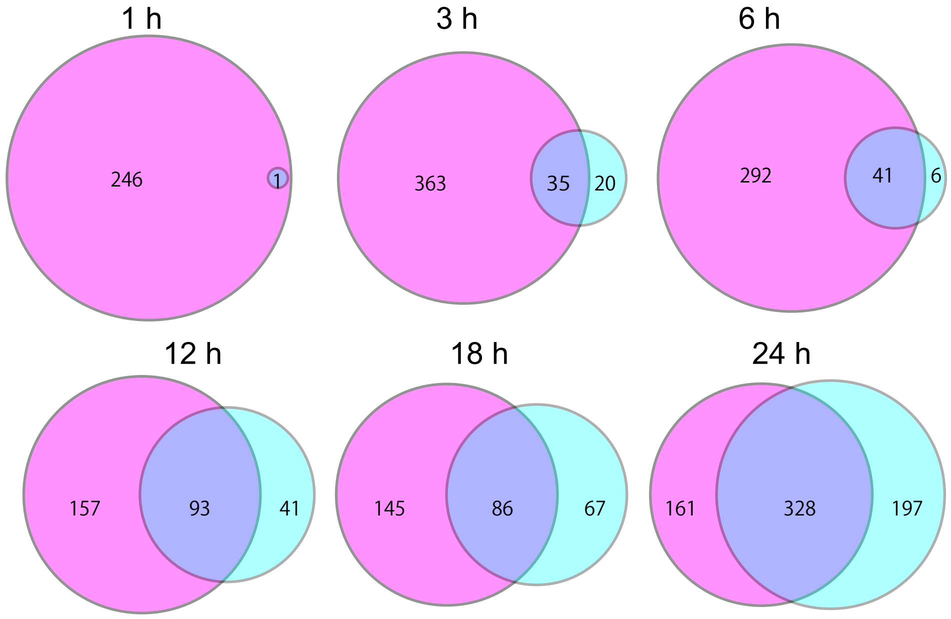

Supplemental Fig. 4. Number of genes downregulated at each time point after DMBQ or SyA treatments.

A. Venn diagrams show the number of genes downregulated by DMBQ or SyA treatment at each time point. Differentially expressed genes were selected by edgeR based on the comparison of HIF treatment with water control.

A

Gene ID	Functional annotation
gene-SHERM_06172	Cytokinin hydroxylase
gene-SHERM_15393	Pirin-like protein
gene-SHERM_06785	Transcription factor bHLH93
gene-SHERM_18147	Pirin-like protein
gene-SHERM_20979	Regulatory protein NPR3
gene-SHERM_11299	Transcription factor bHLH93
gene-SHERM_03094	Probable NAD(P)H dehydrogenase (quinone) FQR1-like 1
gene-SHERM_20353	cytochrome P450- family 81- subfamily D-polypeptide 8
gene-SHERM_01797	Cytochrome b561 and DOMON domain-containing protein
gene-SHERM_10416	Unknown protein
gene-SHERM_24153	ABC transporter B family member 15

B

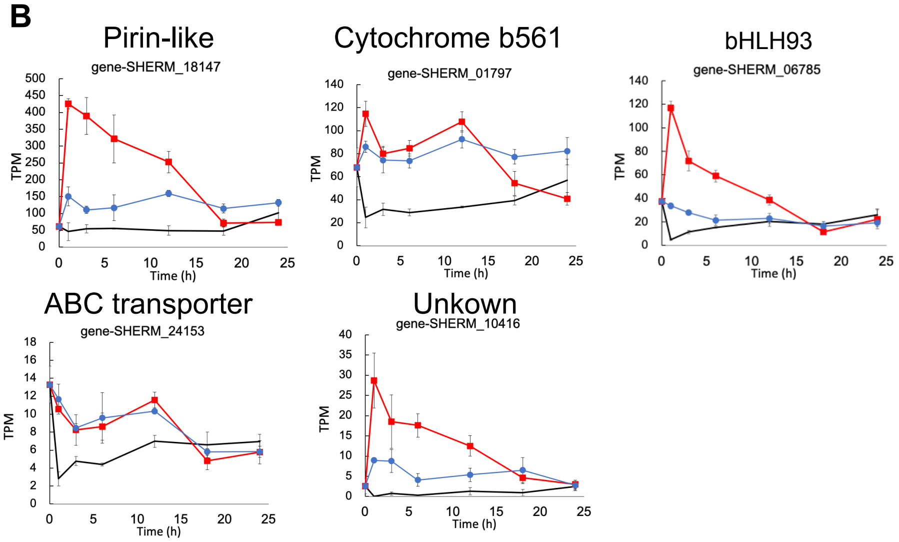

Supplemental Fig. 5.The early core genes commonly induced by DMBQ and SyA at 1 hr after treatments

A. Contigs that are upregulated at 1 hr after treatment of DMBQ and SyA. The top blast hits from public database are shown. B. Expression patterns of early core signaling genes at 1, 3, 6, 12, 18 and 24 h after treatment. The other contigs are shown in Fig. 4B.

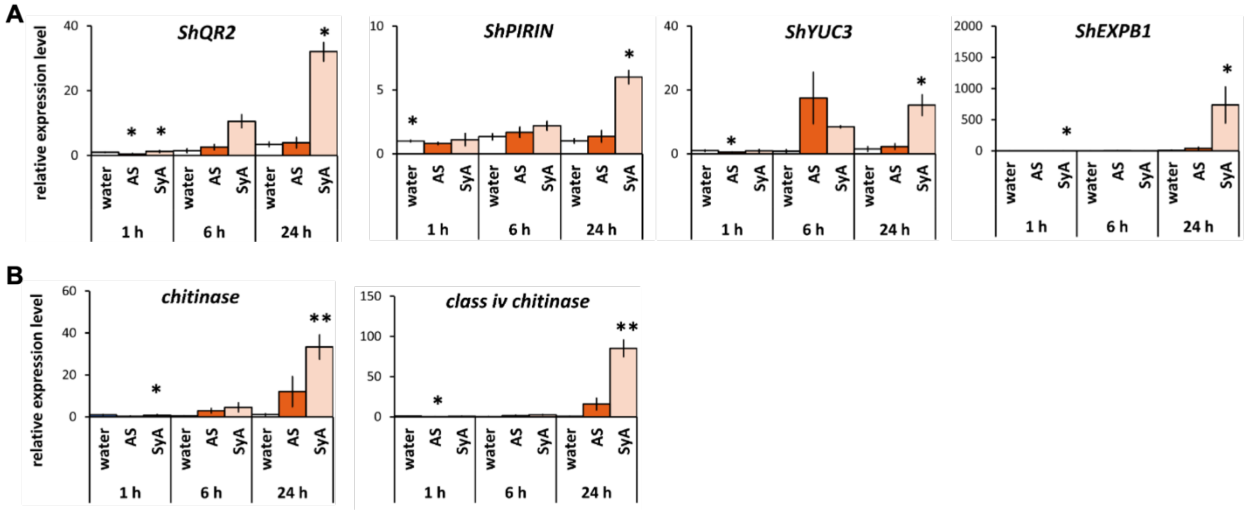

Supplemental Fig. 6. Expression of marker genes in response to phenolics and quinone in *S. hermonthica*. Quantification for the expression of early DMBQ-responsive genes (A) and rice infection-responsive genes (B) in response to 50 μM AS and 20 μM SyA using qRT-PCR. Gene expressions were normalized with water treated control sample at 1 h. Data represents mean±SE with three replicates. Asterisks indicate significant differences from water control (*t*-test, **: p<0.01, *: 0.01<p<0.05).

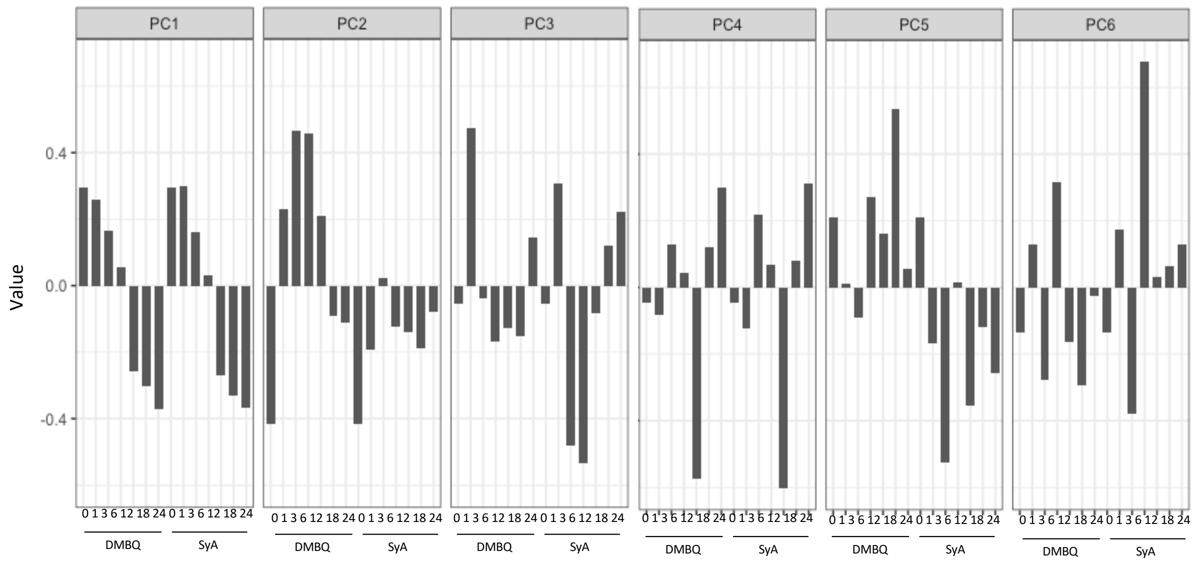

Supplemental Fig. 7. Factor loadings for the genes obtained from transcriptomic experiments. PC1 to PC6 were calculated from samples treated by DMBQ and syringic acid**.**

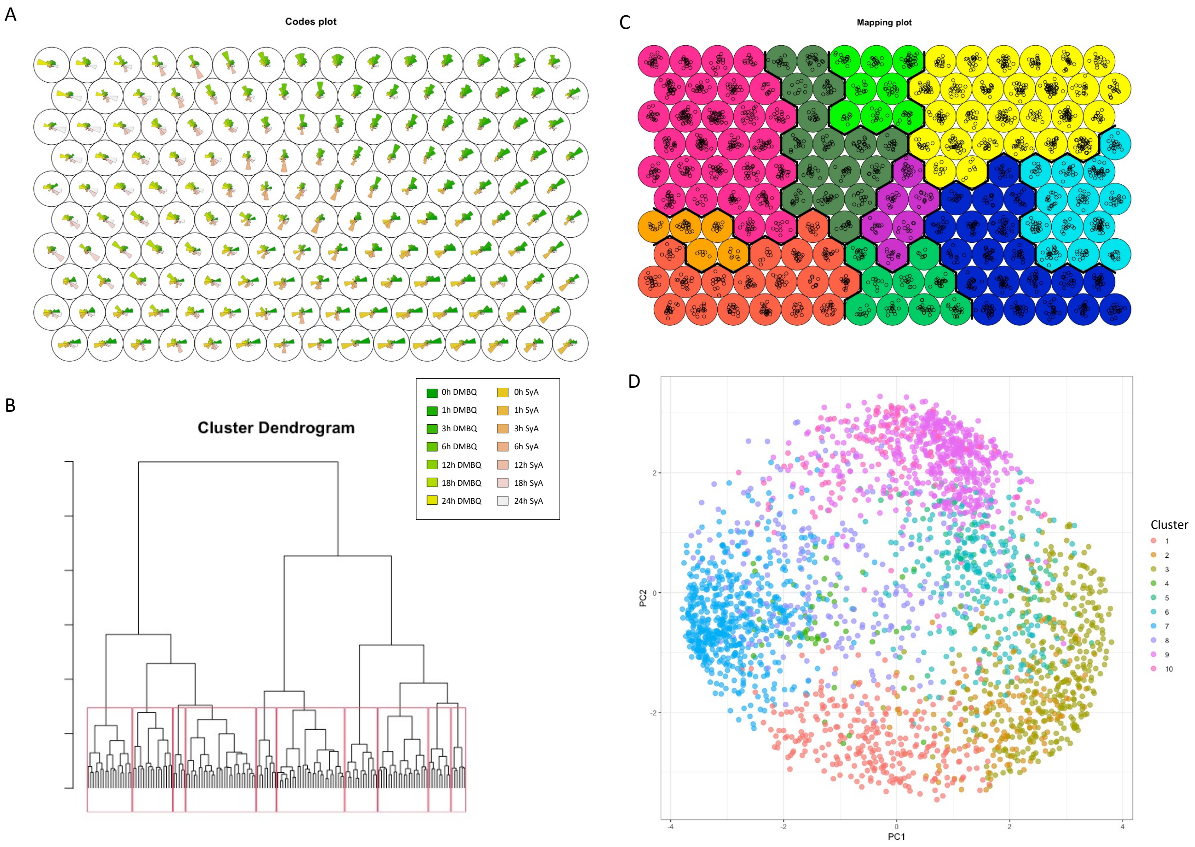

Supplemental Fig. 8. Clustering analysis for differentially expressed genes (DEG). (A) The obtained DEGs were divided into 150 groups based on gene patterns. (B) The relationships among the the 150 groups were displayed as dendrograms. (C) Based on the dendrograms (B), the groups were further divided into 10 groups as indicated by different colors. (D) PCA analysis of the extracted DEGs divided by 10 groups were was performed by SOM cluster analysis.

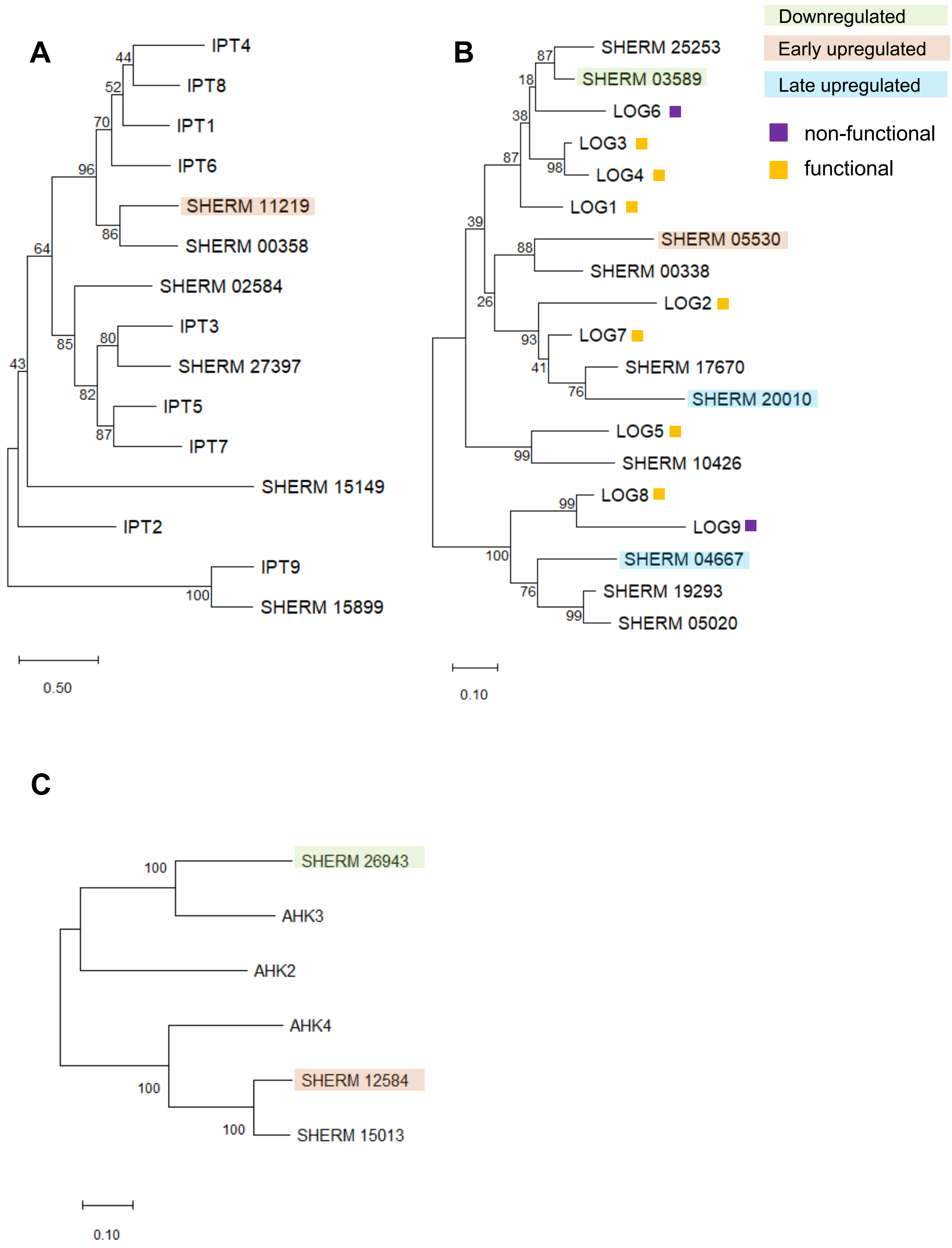
A
IPT4
IPT8
IPT1
IPT6
SHERM 11219
SHERM 00358
SHERM 02584
IPT3
SHERM 27397
IPT5
IPT7
SHERM 15149
IPT2
IPT9
SHERM 15899
0.50
B
SHERM 25253
SHERM 03589
LOG6
LOG3
LOG4
LOG1
SHERM 05530
SHERM 00338
LOG2
LOG7
SHERM 17670
SHERM 20010
LOG5
SHERM 10426
LOG8
LOG9
SHERM 04667
SHERM 19293
SHERM 05020
0.10
Downregulated
Early upregulated
Late upregulated
non-functional
functional
C
SHERM 26943
AHK3
AHK2
AHK4
SHERM 12584
SHERM 15013
0.10

C

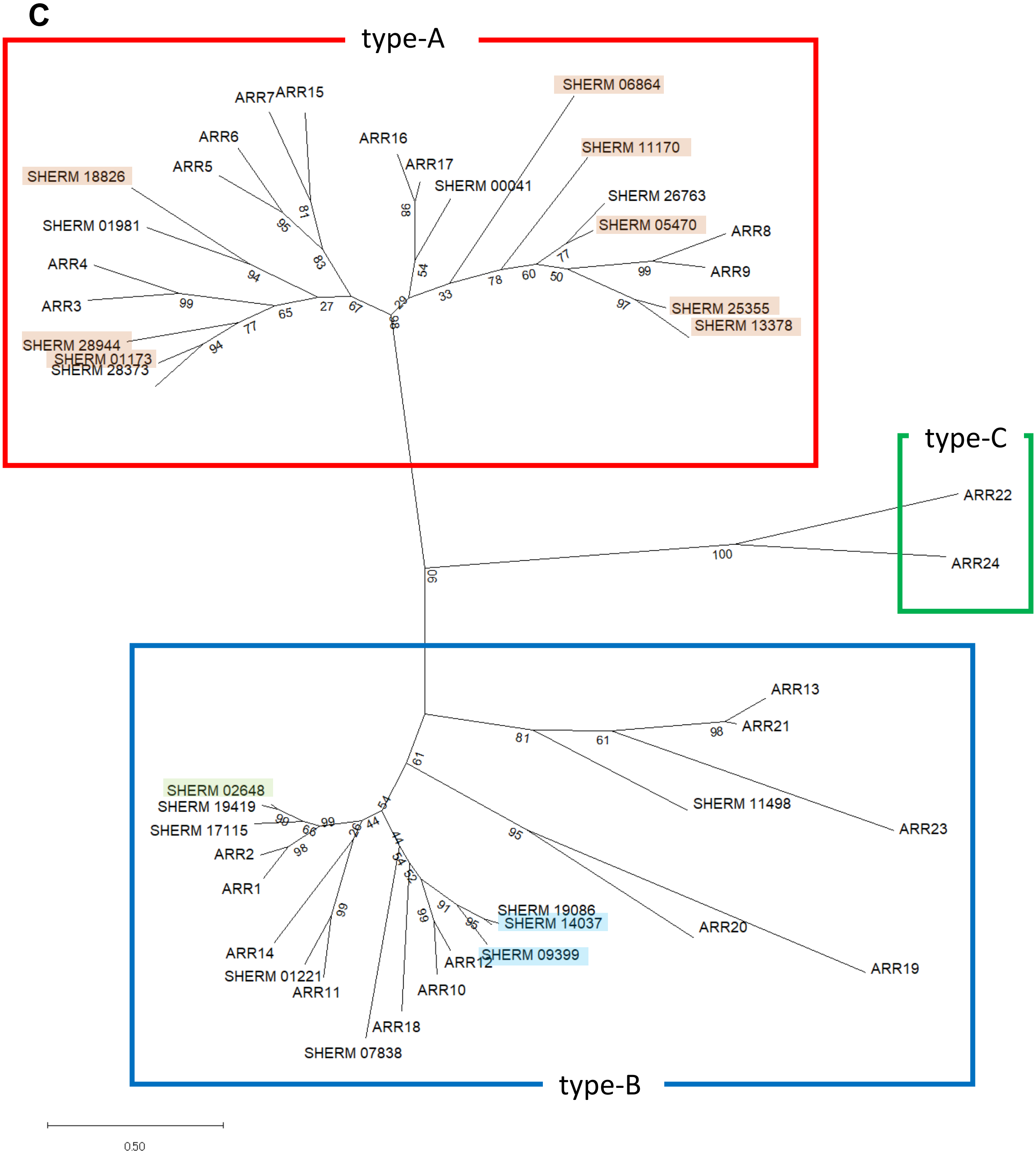

Supplemental Fig. 9. Phylogenetic trees of CK biosynthesis and signaling genes in *S. hermonthica* and *A. thaliana.*

The phylogenetic trees for CK biosynthesis proteins, IPT (A) and LOG (B), HK (C) and ARR (D)were inferred based on amino acid sequences using maximum likelihood (ML) method. The bootstrap values are shown on each node. The expression patterns are indicated by the color highlight. The functional or non-functional LOGs according to Kuroha et al. 2006 are indicated by square marks.
